# Identification of ophiostomatalean fungi associated with *Tomicus pilifer* infesting *Pinus koraiensis* in Northeastern China

**DOI:** 10.3389/fmicb.2022.919302

**Published:** 2022-09-02

**Authors:** Huimin Wang, Caixia Liu, Fangzheng Yue, Dong-Hui Yan, Quan Lu

**Affiliations:** ^1^Key Laboratory of Forest Protection of National Forestry and Grassland Administration, Ecology and Nature Conservation Institute, Chinese Academy of Forestry, Beijing, China; ^2^Biological Disaster Control and Prevention Center, National Forestry and Grassland Administration, Shenyang, China

**Keywords:** *Ceratocystiopsis*, *Graphilbum*, *Leptographium*, *Ophiostoma*, taxonomy

## Abstract

Ophiostomatalean fungi usually facilitate bark beetles to infest tree hosts and seriously endanger the health of coniferous forests. *Tomicus pilifer* Spessivtsev is a common endemic bark beetle in Asia and primarily threatens *Pinus koraiensis*. *Tomicus* species have similar morphology; however, they can be differentiated by their genetic characteristics through phylogenetic analyses. To date, the 28S rDNA sequence of *T. pilifer* and the diversity of ophiostomatalean fungi associated with *T. pilifer* have not been reported. In this study, we aimed to clarify the taxonomic status of *T. pilifer* and identify ophiostomatalean fungi associated with *T. pilifer* infesting *P. koraiensis* in northeastern China. In total, 315 ophiostomatalean fungal strains were isolated from 62 adults of *T. pilifer* and 220 tissue samples from *T. pilifer* galleries in Jilin Province. Thirty-five representative strains were further identified by comparing their morphological and physiological characteristics and conducting the phylogenetic analysis of ITS, ITS2-LSU, TUB2, and TEF1-α. We identified nine species of ophiostomatalean fungi belonging to four genera, which included six novel species (*Ceratocystiopsis changbaiensis* sp. nov., *Leptographium linjiangense* sp. nov., *Leptographium qieshaoense* sp. nov., *Ophiostoma piliferi* sp. nov., *Ophiostoma tonghuaense* sp. nov., and *Ophiostoma yaluense* sp. nov.), two previously described species (*Graphilbum interstitiale* and *Ophiostoma fuscum*), and one undefined specie (*Ceratocystiopsis* sp. 1). To the best of our knowledge, this is the first report of *G*. *interstitiale* and *O*. *fuscum* in China and the fungal diversity of ophiostomatalean in *T. pilifer*. The dominant species were *O. piliferi* and *L. qieshaoense*, representing 39.37% and 35.87% of the isolates, respectively. The results of this study provide valuable information on the symbiotic relationship between bark beetles and ophiostomatalean fungi.

## Introduction

Synergetic infections of forest insects and microbes are key drivers of deforestation worldwide (Wingfield et al., 2010, 2016; Six and Wingfield, 2011; Linnakoski et al., 2012a). Microbial pathogens and insects can cause more damage synergistically than when acting alone (Raffa et al., 2008). Some well-known examples of synergetic infections that cause major forest disturbances are those of ophiostomatalean fungi and beetles (Grégoire et al., 2015).

The pine shoot beetle, *Tomicus* Latreille (syn. Blastophagus Eichhoff, Myelophilus Eichhoff, Scolytidae, Coleoptera), includes eight species; some of these kill conifers in Eurasian pine forests (Kirkendall et al., 2008; Li et al., 2010; Lieutier et al., 2015). *Tomicus* is considered one of the most aggressive bark beetles (Lieutier et al., 2015). During its life cycle, it can invade both sapwood and branches and infest multiple hosts. Currently, seven species have been recorded in China (Wang et al., 2019). *Tomicus pilifer* Spessivtsev, also called the Korean pine shoot beetle, is endemic to Asia (Russia and China) and primarily lives on *Pinus koraiensis* (Seib. Ex Succ.). However, it has also been documented on other Asian pines, such as *P. armandii, P. tabulaeformis*, and *P. yunnanensis* (Lieutier et al., 2015). In China, *T. pilifer* is mainly distributed in the Jilin and Gansu provinces, and 90% of the damaged trees are *P. koraiensis*. Moreover, *Tomicus pilifer* primarily infests the new shoots of *P. koraiensis*, which results in the withering of branches and leaves, hindered tree development, and death of seriously damaged trees (Wang et al., 2000).

*Tomicus* species have highly similar morphology, making it difficult to distinguish them based on morphological traits alone (Lieutier et al., 2015). For example, *T. destruens* and *T. yunnanensis* were previously considered to be *T. piniperda;* however, molecular studies revealed that they are two separate species (Gallego and Galián, 2001; Kerdelhué et al., 2002; Kohlmayr et al., 2002; Duan et al., 2004). Similarly, *T. armandii* was classified as a new species based on molecular and morphological data (Li et al., 2010). Although 28S rDNA phylogenetic analysis showed a genetically well-characterized clade, including six *Tomicus* species, it did not include *T. pilifer* and *T. puellus* (Li et al., 2010).

Fungi in the order Ophiostomatales (Ascomycota) are referred to as ophiostomatalean fungi. Ophiostomatalean fungi are the most common fungi associated with bark and ambrosia beetles (Wingfield et al., 1993; De Beer and Wingfield, 2013). They have been documented to assist beetles to overcome host defenses (Lieutier et al., 2009; DiGuistini et al., 2011); providing nutrition for beetles to complete their life cycle (Ayres et al., 2000; Lee et al., 2005, 2006; Bleiker and Six, 2007); assisting them in pheromone production; and protecting them from detrimental fungi (Lu et al., 2011; Cale et al., 2019). For example, *Leptographium yunnanense* is the most virulent fungus associated with *T. yunnanensis* and it can produce pathogenic toxins after synergetic infections of the host with bark beetles (Liao and Ye, 2004). Moreover, *Leptographium wingfieldii* is a strong pathogenic fungus associated with *T. piniperda* and was discovered to be able to kill 87% of *Pinus sylvestris* seedlings 4 weeks after inoculation (Jankowink, 2006). In addition, *Ophiostoma tingens* are nutritionally fungal, as the food for the larvae of *Tomicus minor* (Kirisits, 2004).

Ophiostomatalean fungi associated with *Tomicus* spp. have been reported earlier (Lieutier et al., 2015; Chang et al., 2017; Wang et al., 2019). A total of 52 ophiostomatalean fungi are associated with *Tomicus* species (Supplementary Table S1). *Tomicus piniperda* and *T. minor* are the most widely distributed species, and the diversity of their associated ophiostomatalean fungi is greater than that of other *Tomicus* species, with 30 (Jacobs et al., 2003; Zhou et al., 2004; Kim et al., 2005; Jankowiak, 2006; Jankowink, 2006; Lieutier et al., 2015) and 27 (Jankowiak, 2006; Jankowink, 2006; Linnakoski et al., 2010; Wang et al., 2019; Pan et al., 2020b) species located in Eurasia, respectively. A comparative and systematic study was conducted on ophiostomatalean fungi associated with *T. yunnanensis* and *T. brevipilosus*. At present, 15 (Zhou et al., 2000, 2013; Chang et al., 2017; Wang et al., 2019; Pan et al., 2020a,b) and 3 (Chang et al., 2017; Wang et al., 2019) species have been reported in Asia, respectively. *Ophiostoma minus* was documented as the dominant species associated with *T. piniperda* in Europe and Japan, and with *T. yunnanensis* in China (Wang et al., 2019). However, its internal transcribed spacer (ITS) region and *Bacillus thuringiensis* (BT) integrated genes were found to differ, with *O. minus* strains obtained from *T. yunnanensis* and *T. piniperda* being classified into two different clades. In addition, *Ophiostoma canum* and *Ophiostoma brevipilosi* were frequently isolated in association with *T. minor* and *T. brevipilosus*, respectively (Wang et al., 2019). *Tomicus destruens* is only distributed in the Mediterranean region of Europe (Lieutier et al., 2015), and only six ophiostomatalean fungi have been sporadically recorded to be associated with it. In addition, three ophiostomatalean fungi have been reported to be associated with *T. armandii* in China (Yin et al., 2015; Pan et al., 2018, 2020a,b). However, ophiostomatalean fungi associated with *T. pilifer* and *T. puellus* have not been reported.

In this study, we elucidated the diversity of ophiostomatalean fungi associated with *T. pilifer* and its galleries in infested *P. koraiensis* in northeastern China. Phylogenetic analyses of 28S rDNA datasets were also used to identify the affiliation of *T. pilifer* with *Tomicus* spp. This is the first report of ophiostomatalean fungal diversity in *T. pilifer*, and the results of this study provide a valuable theoretical basis for understanding the mechanisms of *T. pilifer* infestations.

## Materials and methods

### Sample collection and fungus isolation

Adult *Tomicus pilifer* and their bark galleries were sampled in Linjiang (N: 126°55'81″ E: 41°49″53″) and Tonghua (N: 125°45'28″ E: 41°40″34″), Jilin Province, northeast China from June to July 2020. *Pinus koraiensis* sampled in this study showed signs of being dead or dying. Adult beetles were individually placed in Eppendorf tubes, and their galleries were placed individually in envelope bags. Both beetles and galleries were stored at 5°C until fungal isolation. Each beetle was dismembered directly without superficial disinfection and transferred onto a 2% agar medium (20 g Biolab agar and 1,000 mL deionized water). The galleries were cut into approximately 5 × 5 mm tissue pieces, disinfected with 1.5% sodium hypochlorite (NaClO) for 1 min, rinsed with sterile water three times, and placed in 9-cm Petri dishes with 2% agar medium. All cultures were incubated in humid chambers in the dark at 25°C and inspected every day for mycelial mass. The mycelium apex was used to purify all the strains. Pure strains were transferred onto 2% malt extract agar (MEA; 20 g Biolab malt extract, 20 g Biolab agar, and 1,000 mL deionized water). All strains used in this study were deposited in the culture collection of the Forest Pathology Laboratory of the Chinese Academy of Forestry (CXY). Specimen were deposited in the China Forestry Culture Collection Center (CFCC: part of the National Infrastructure of Microbial Resources; Table 1).

**Table 1:** Representative strains of ophiostomatalean fungi associated with *Tomicus pilifer* infesting *Pinus koraiensis* obtained in northeastern China.

**Taxon**	**Species^a^**	**CFCC No.^b^^,^ ^d^**	**CXY No.^c^**	**Location**	**GenBank number** ^**e**^		
					**ITS/ITS2-LSU**	**TUB2**	**EF1-α**
1	***Ceratocystiopsis***	57411T	4031	Linjiang	OM397400	OM628691	–
	***changbaiensis*** **sp. nov**.	57453	4032	Linjiang	OM397401	OM628692	–
2	*Ceratocystiopsis* sp. 1	57454	4033	Linjiang	OM397402	OM568747	–
3	*Graphilbum interstitiale*	57329	4034	Linjiang	OM397403	OM524677	OM524681
		57340	4035	Linjiang	OM397404	OM524678	OM524682
		57387	4036	Linjiang	OM397405	OM524679	OM524683
		5733	4037	Linjiang	OM397406	OM524680	OM524684
4	***Leptographium linjiangense*** **sp. nov**.	57337T	4044	Linjiang	OM388433	OM643329	OM687498
		57338	4045	Linjiang	OM388434	OM643330	OM687499
		57339	4046	Linjiang	OM388435	OM643331	OM687500
5	***L. qieshaoense*** **sp. nov**.	57328T	4038	Linjiang	OM388427	OM643332	OM687501
		57327	4039	Linjiang	OM388428	OM643333	OM687502
		57343	4040	Tonghua	OM388429	OM643334	OM687503
		57345	4041	Linjiang	OM388430	OM643335	OM687504
		57342	4042	Tonghua	OM388431	OM643336	OM687505
		57344	4043	Linjiang	OM388432	OM643337	OM687506
6	*Ophiostoma fuscum*	57379	4060	Linjiang	–	OM475644	–
		57369	4061	Tonghua	OM397407	OM475645	–
		57373	4062	Linjiang	–	OM475646	–
		57408	4063	Linjiang	OM397408	OM475647	–
		57409	4064	Linjiang	–	OM475648	–
		57383	4065	Linjiang	–	OM475649	–
7	***O. piliferi*** **sp. nov**.	57410	4047	Linjiang	–	OM678560	OM718776
		57370T	4048	Linjiang	OM397409	OM678561	OM718777
		57371	4049	Linjiang	–	OM678562	OM718777
		57377	4050	Tonghua	OM397410	OM678563	OM718779
		57376	4051	Tonghua	OM397411	OM678564	OM718780
		57372	4052	Tonghua	–	OM678565	OM718781
8	***O. tonghuaense*** **sp. nov**.	57378	4053	Linjiang	–	OM678566	OM718782
		57375	4054	Linjiang	OM397413	OM678567	OM718783
		57374T	4055	Linjiang	OM397412	OM678568	OM718784
		57384	4056	Linjiang	–	OM678569	OM718785
9	***O. yaluense*** **sp. nov**.	57381	4057	Linjiang	OM397415	OM678570	OM718786
		57380	4058	Linjiang	–	OM678571	OM718787
		57382T	4059	Tonghua	OM397414	OM678572	OM718788

a Species names in bold are novel species described in this study.

b CFCC: China Forestry Culture Collection Center, Beijing, China.

c CXY (Culture Xingyao): Culture collection of the Ecology and Nature Conservation Institute, Chinese Academy of Forestry.

d T = ex-holotype isolate.

e ITS: the internal transcribed spacer regions 1 and 2 of the nuclear ribosomal DNA operon, including the 5.8S region; ITS2-LSU: the internal transcribed spacer 2 and part of the 28S of the rDNA operon; TUB2: the β-tubulin gene region (TUB2); TEF1-α: the transcription elongation factor 1-α gene region.

Sequences missing data are indicated by “–”.

### Morphological and physiological characteristics

Beetle specimens were examined using an Olympus SZX16 stereomicroscope (Olympus, Tokyo, Japan). Based on their overall morphology, the beetles were taxonomically identified using the guidelines established by Lieutier et al. (2015). Morphological descriptions were made with strains cultivated on 2% MEA. Pure cultures were incubated for 7–15 days at 25°C in the dark. A Zeiss Axiocam 506 color digital camera (Carl Zeiss Ltd., Munich, Germany) and an Olympus BX51 (Olympus, Center Valley, PA, United States) microscope with differential interference contrast illumination were used to observe the culture morphology and reproductive structures of the fungi. Fifty measurements were repeated for each reproductive structure and are presented as (minimum–) (mean - standard deviation) (mean standard deviation) (–maximum). All data for the type specimens were deposited in MycoBank.

The optimal growth temperatures of the representative strains were determined in the dark at temperatures ranging from 5–35°C at 5°C intervals. A 5-mm-diameter agar plug from an actively growing margin of fungal colonies was set upside down in the center of a 90-mm-diameter 2% MEA plate, with five replicate plates. Colony colors on the surface and reverse side were assessed according to the color charts of Rayner (1970).

### DNA extraction, amplification, and sequencing

The mycelia were collected from the actively growing colony margin of each representative strain and transferred to 2-mL Eppendorf tubes. Total genomic DNA of *T. pilifer* and fungi were extracted and purified using an Invisorb Spin Cell Mini Kit (DP304, Tiangen, Beijing, China) and an Invisorb Spin Plant Mini Kit (DP305, Tiangen), respectively, following the manufacturer's protocol.

The primer pair 28Sscol1/D3R was used to amplify the 28S rDNA of *T. pilifer* (Li et al., 2010). Four primer pairs were used for fungal nucleotide polymerase chain reaction (PCR) amplification and sequencing. The ITS1-F/ITS4 primer pair (White et al., 1990; Gardes and Bruns, 1993) was used to amplify the internal transcribed spacer (ITS) regions (ITS1 and ITS2, including the 5.8S gene); ITS3/LR3 (White et al., 1990) was used for the internal transcribed spacer 2 and part of the 28S of the rDNA operon (ITS2-LSU); Bt2a/Bt2b (Glass and Donaldson, 1995) was used for the parts of the β-tubulin (TUB2) gene; and EF1F/EF2R (Jacobs et al., 2004) was used to amplify the elongation factor 1-α (TEF1-α) gene region.

The polymerase chain reaction (PCR) mixtures had a volume of 25 μL (10 × EasyTaq Buffer [TianGen], 50 μM dNTPs, 0.1 μM of each primer, 0.75 U Taq DNA polymerase, and 1–10 ng genomic DNA), and the reactions were conducted using a thermocycler (Applied Biosystems, Foster City, CA, USA), following the manufacturer's instructions. The PCR conditions were similar to those described in the literature for primer design. PCR products were sequenced by bidirectional sequencing using a CEQ 2000 XL capillary automated sequencer (Beckman Coulter, Brea, CA, USA), and BioEdit v 7.2.0. was used for analyzing splicing patterns.

### Phylogenetic analyses

Preliminary BLAST searches of the obtained ITS DNA sequences were performed in the NCBI GenBank database, and closely related authentic sequences were downloaded for further phylogenetic analyses. Alignments of the sequences were performed with MAFFT v. 7 (http://mafft.cbrc.jp/alignment/server/) using the iterative refinement method (FFT-NS-i strategy with a 200 PAM/k = 2 scoring matrix) and edited manually using MEGA v. 7.0.26. Maximum likelihood (ML) and Bayesian inference (BI) methods were implemented. Gaps were treated as the fifth character.

ML phylogenetic analyses were performed using RAxML v. 7.0.3, (Stamatakis, 2006) with the GTR-GAMMA model. Approximately 1,000 bootstrap replicates were conducted to assess the overall reliability of tree topology and bootstrap values. BI phylogenetic analyses were performed using the Markov chain Monte Carlo (MCMC) algorithm in MrBayes v. 3.1.2 (Ronquist and Huelsenbeck, 2003). Four MCMC chains were run simultaneously from a random starting tree for 5,000,000 generations, and trees were sampled every 100 generations to calculate posterior probabilities. The first 25% of the sampled trees were discarded as burn-in, and the remaining trees were used to construct majority-rule consensus trees. The topology of the phylogenetic trees was subsequently edited using FigTree v.1.4.2 and Adobe Illustrator CS6.

## Results

### Sample collection and isolation

In this study, 194 and 89 *T. pilifer* individuals were collected from *P. koraiensis* in Linjiang and Tonghua, Jilin Province, respectively. A total of 315 ophiostomatalean fungal strains were obtained from 62 adults and 220 tissue pieces of *T. pilifer* galleries at the two sites. Of these, 256 and 59 ophiostomatalean strains were isolated from *T. pilifer* and its galleries, respectively (Table 2). Moreover, 237 strains were obtained from 43 adults and 220 tissue pieces at Linjiang, and 78 strains were obtained from 19 adults at Tonghua.

**Table 2:** Diversity and abundance of the isolated ophiostomatalean strains.

**Taxon**	**Genus**	**Species**	**Numbers of isolates**		**Total**	**Total percentage**
			**Beetles**	**Galleries**		
1	*Ceratocystiopsis*	*C. changbaiensis*	2	0	2	0.63%
2		*Ceratocystiopsis* sp. 1	1	0	1	0.32%
3	*Graphilbum*	*Gra. interstitiale*	9	17	26	8.25%
4	*Leptographium*	*L. linjiangense*	9	3	12	3.81%
5		*L. qieshaoense*	104	9	113	35.87%
6	*Ophiostoma*	*O. fuscum*	3	9	12	3.81%
7		*O. piliferi*	106	18	124	39.37%
8		*O. tonghuaense*	17	2	19	6.03%
9		*O. yaluense*	5	1	6	1.90%
	Total		256	59	315	100.00%

### Phylogenetic analyses

The ML and BI phylogenetic methods used in this study obtained concordant topologies with marginal discrepancies in node support values. Three *T. pilifer* adults were selected as representative bark beetles for the phylogenetic analyses of *Tomicus* based on 28S rDNA datasets (GenBank Accession No.: TOMP1 = ON042458, TOMP2 = ON042459, TOMP3 = ON042460). The 35 representative strains were separated into four genera (*Ceratocystiopsis, Graphilbum, Leptographium*, and *Ophiostoma*) and nine species (Taxa 1–9) based on DNA fragment analyses of ITS, ITS2-LSU, TUB2, and TEF1-α, revealing six new species, two known species, and one undefined species.

**Figure 1:**
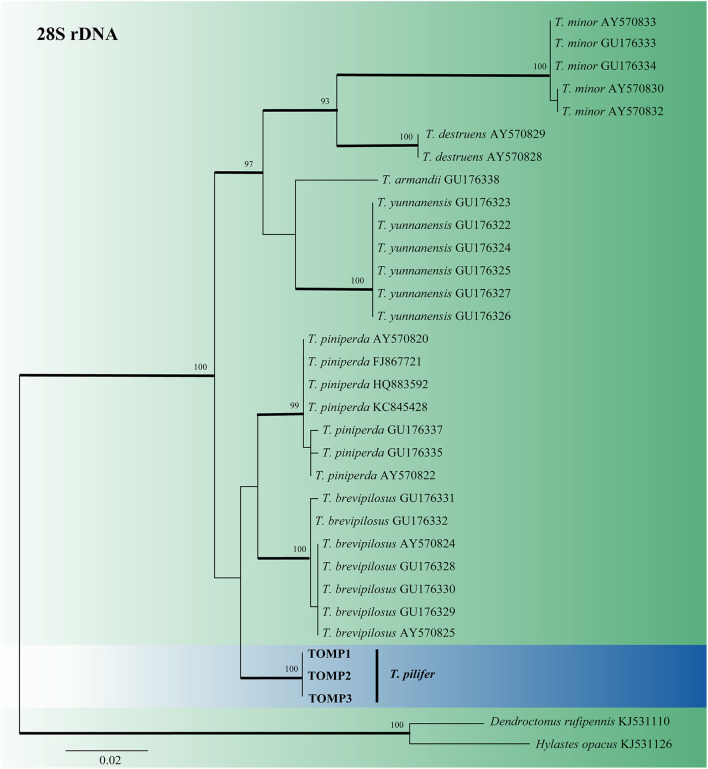
ML tree of *Tomicus* generated from the 28S rDNA sequence data. Sequences of *T. pilifer* obtained in this study are presented in bold typeface. The bold branches indicate posterior probability values >0.9. Bootstrap values of ML ≥ 70% are recorded at the nodes. The final alignment of 566 positions, including gaps.

For *Tomicus*, the 28S rDNA sequences of six known pine shoot beetles and *T. pilifer* were compiled and analyzed (Figure 1). The 28S rDNA phylogenetic tree confirmed that the three *T. pilifer* sequences formed independent, well-supported clades that were closely related to *T. piniperda* and *T. brevipilosus*. The phylogenetic tree also showed that the seven known *Tomicus* species were divided into two large branches: *T. pilifer, T. piniperda*, and *T. brevipilosus* clustered into one branch, and *T. armandii, T. destruens, T. minor*, and *T. yunnanensis* clustered into another.

**Figure 2:**
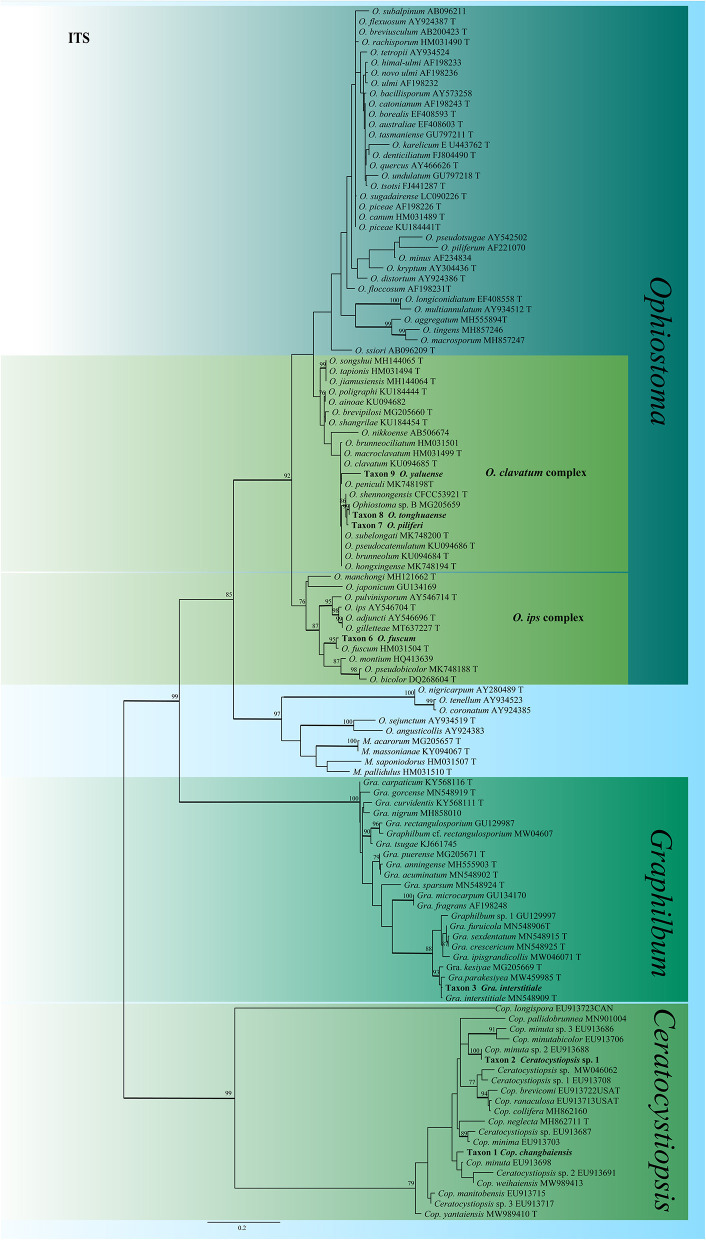
ML tree of *Ceratocystiopsis, Graphilbum* and *Ophiostoma* generated from the ITS sequence data. Novel sequences obtained in this study are presented in bold typeface. The bold branches indicate posterior probability values >0.9. Bootstrap values of ML ≥ 70% are recorded at the nodes. T, ex-type strains. The final alignment of 662 positions, including gaps.

**Figure 3:**
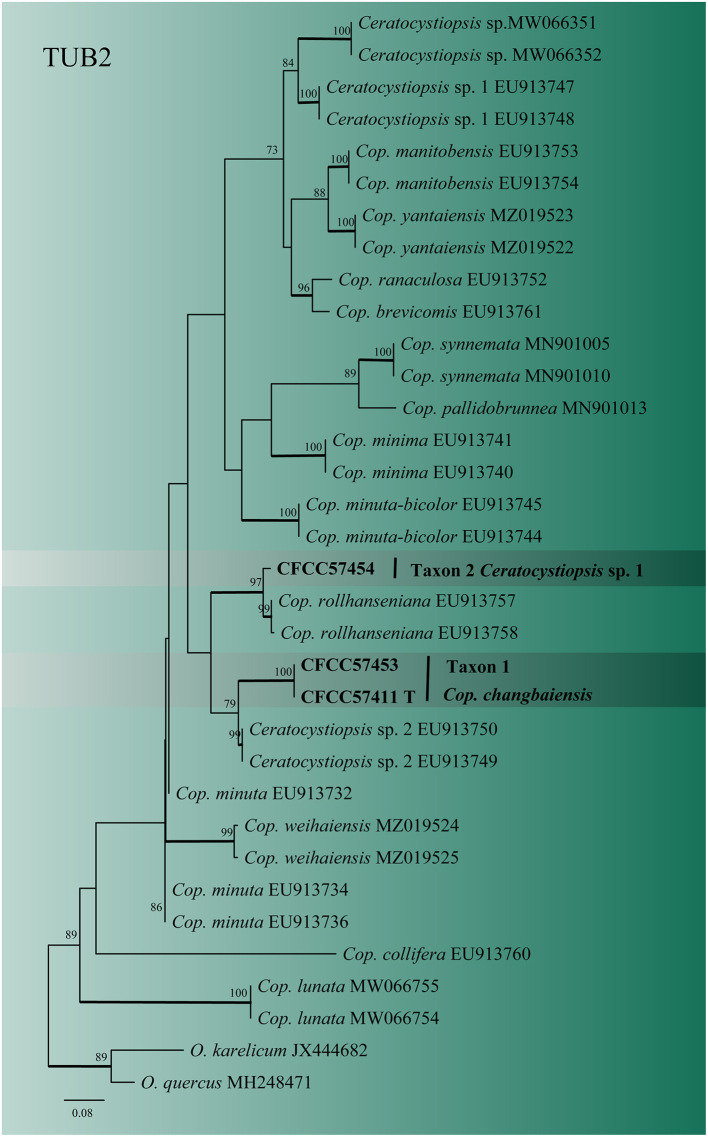
ML tree of *Ceratocystiopsis* generated from the TUB2 (Taxon 1, 2) sequence data. Novel sequences obtained in this study are presented in bold typeface. The bold branches indicate posterior probability values >0.9. Bootstrap values of ML ≥ 70% are recorded at the nodes. T, ex-type strains. The final alignment of 471 positions, including gaps.

For *Ceratocystiopsis*, the three representative strains formed two distinct clades (Figures 2, 3) based on the ITS and TUB2 datasets. In the ITS and TUB2 trees, Taxon 1 (consisting of two strains) formed a well-supported clade, closely related to *Ceratocystiopsis* sp. 2. Taxon 2 (single strain) was closest to, but clearly distinct from, *Cop. rollhanseniana*. Both ITS and TUB2 phylogenetic analyses confirmed that Taxa 1 and 2 represented undescribed species.

In *Graphilbum*, the results from ITS, TUB2, and TEF1-α sequences suggested that Taxon 3 (consisting of four strains) emerged as conspecific to *Gra. interstitiale* (Figure 2 and Supplementary Figures S1, S2).

**Figure 4:**
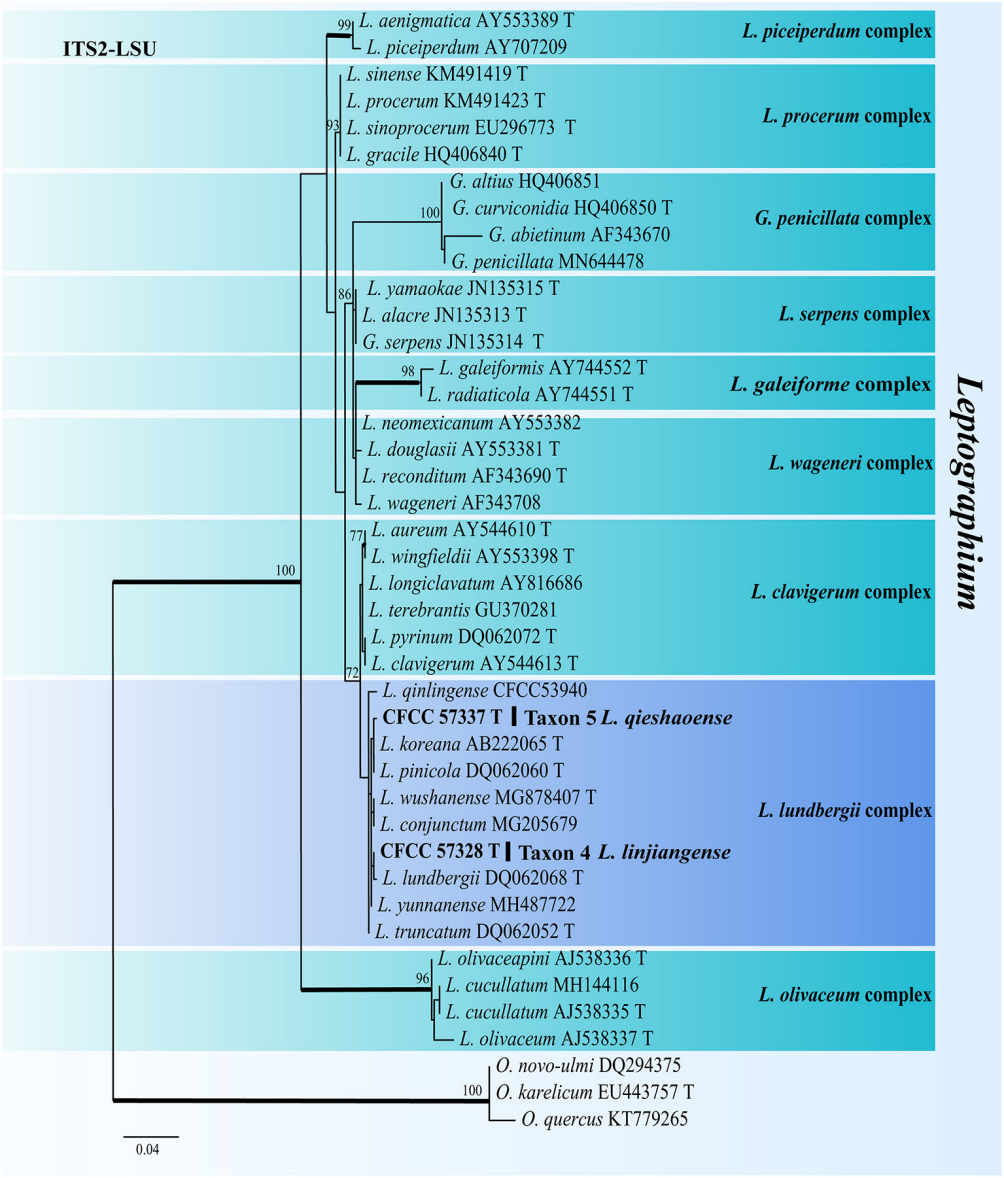
ML tree of *Leptographium* generated from the ITS2-LSU (Taxon 4, 5) sequence data. Novel sequences obtained in this study are presented in bold typeface. The bold branches indicate posterior probability values >0.9. Bootstrap values of ML ≥ 70% are recorded at the nodes. T, ex-type strains. The final alignment of 597 positions, including gaps.

**Figure 5:**
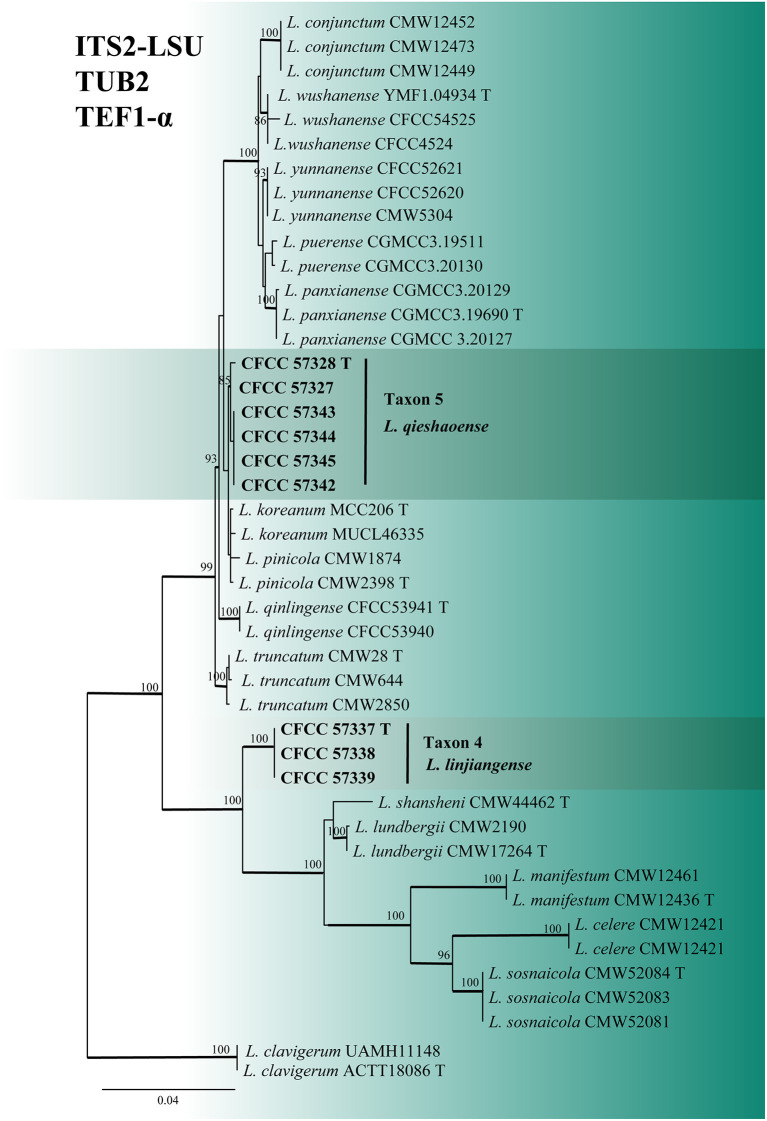
ML tree of *L. lundbergii* complex generated from the combined (ITS2-LSU + TUB2 + EF1-α) (Taxon 4, 5) sequence data. Novel sequences obtained in this study are presented in bold typeface. The bold branches indicate posterior probability values >0.9. Bootstrap values of ML ≥ 70% are recorded at the nodes. T, ex-type strains. The final alignment of 1597 positions, including gaps.

For the phylogenetic analyses of *Leptographium*, both the ITS2-LSU, TUB2, TEF1-α and combined (ITS2-LSU + TUB2 + TEF1-α) datasets confirmed that Taxon 4 (consisting of three strains) and Taxon 5 (consisting of six strains) were nested within the *L. lundbergii* complex (Figures 4, 5). Taxon 4 formed a clade related to, but distinct from, *L. shansheni* and *L. lundbergii* and peripheral to the *L. lundbergii* complex (Figures 4, 5 and Supplementary Figures S3, S4). Taxon 5 formed an independent, well-supported clade with close affinity to *L. koreanum* and *L. pinicola* (Figures 4, 5 and Supplementary Figures S3, S4). Thus, Taxa 4 and 5 represented two undescribed *Leptographium* species.

**Figure 6:**
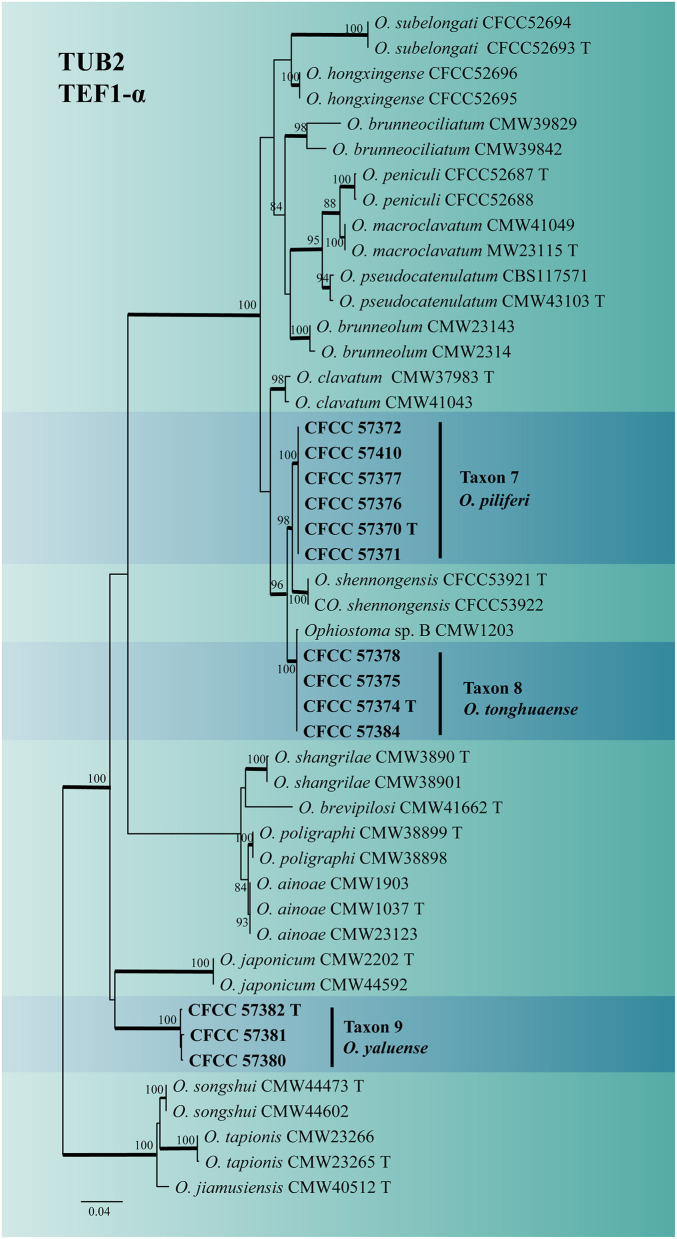
ML tree of *O. clavatum* complex generated from the combined (TUB2 + EF1-α) (Taxon 7, 8, 9) sequence data. Novel sequences obtained in this study are presented in bold typeface. The bold branches indicate posterior probability values >0.9. Bootstrap values of ML ≥ 70% are recorded at the nodes. T, ex-type strains. The final alignment of 1160 positions, including gaps.

The identity of the *Ophiostoma* strains was analyzed using ITS, TUB2, TEF1-α and combined (TUB2 + TEF1-α) datasets. A total of 19 representative strains obtained in this study were separated into four taxa residing in the *O. clavatum* and *O. ips* complex (Figure 2). Taxon 6 (consisting of six strains) was nested in the *O. ips* complex (Figure 2 and Supplementary Figure S7). The ITS and TUB2 dataset analyses confirmed that the taxon was conspecific to *O. fuscum*. Taxa 7–9 nested within the *O. clavatum* complex based on ITS, TUB2, TEF1-α and combined (TUB2 + TEF1-α) dataset analyses. Taxon 7 (consisting of six strains) was positioned in the vicinity of *O. shennongensis* and formed a single, well-supported clade; Taxon 8 (consisting of four strains) together with *Ophiostoma* sp. B (Chang et al., 2017), formed single clades with high node support values. Taxon 9 (consisting of three strains) formed an independent lineage closely related to *O. japonicum*, clearly distinct from other species in the *O. clavatum* complex (Figures 2, 6 and Supplementary Figures S5, S6). These three taxa represented distinct, undescribed species.

### Taxonomy

Based on the phylogenetic analyses, seven of the nine taxa isolated in this study represented undescribed species. However, only one strain was isolated from Taxon 2, and we chose a formal description until more material became available. The remaining six taxa, including one *Ceratocystiopsis* (Taxon 1), two *Leptographium* (Taxa 4 and 5), and three *Ophiostoma* (Taxa 7, 8, and 9) species are described as follows:

#### Ceratocystiopsis changbaiensis H.M. Wang and Q. Lu, sp. nov.

Mycobank MB843471

Etymology: The name refers to the Changbai Mountain, the mountain on which this species was collected.

Type: China. Jilin Province, Linjiang City, adults of *T. pilifer* in *P. koraiensis*. Jun. 2020, H.M. Wang, holotype CXY4031, culture ex-holotype CFCC57411 = CXY4031.

**Figure 7:**
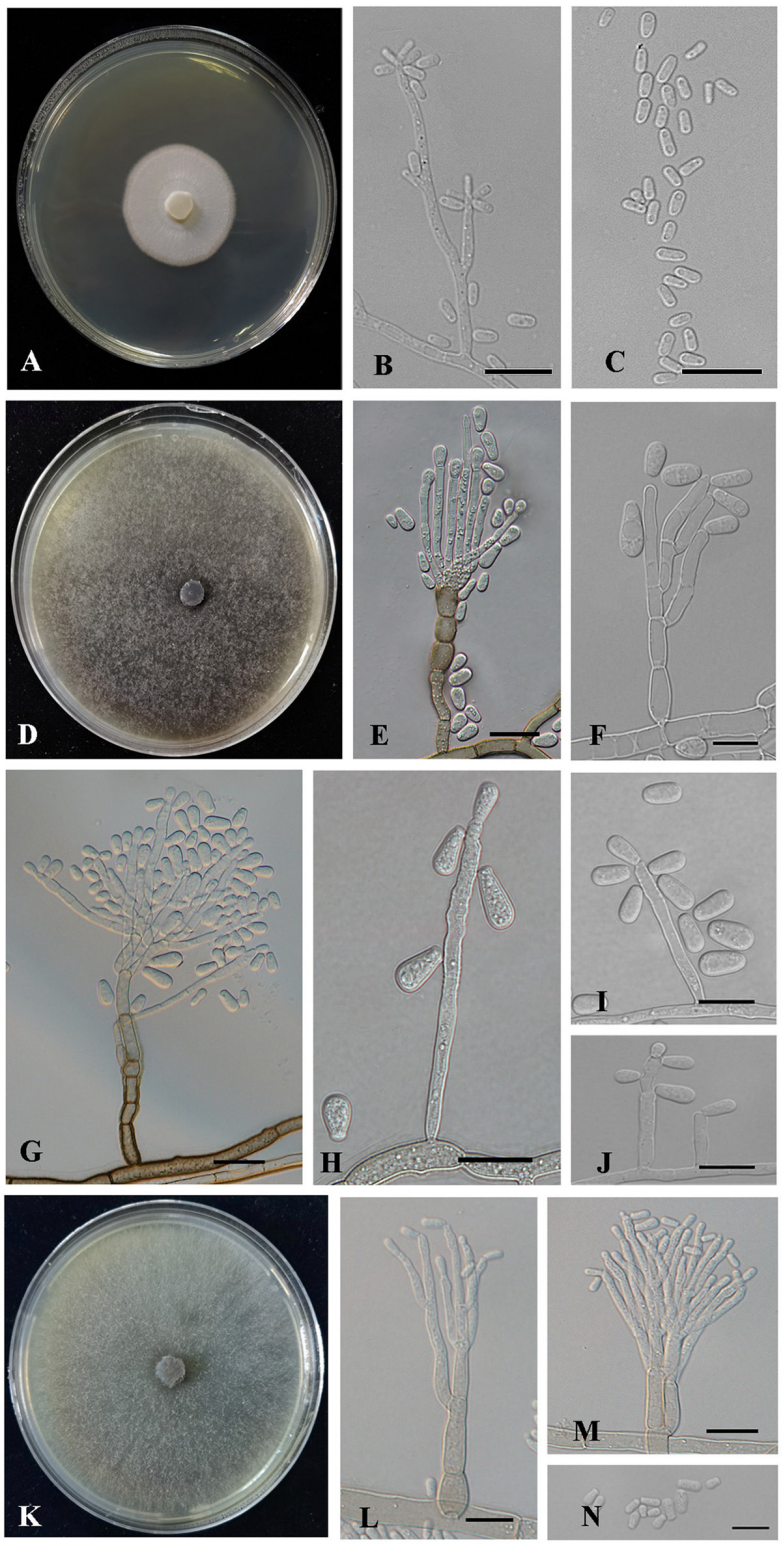
Morphology characteristics of *Ceratocystiopsis* and *Leptographium* new species. **(A–C)** Morphology of *Ceratocystiopsis changbaiensis* sp. nov (Taxon 1). **(A)** Cultures on 2% MEA 10 days after inoculation. **(B,C)** Hyalorhinocladiella-like asexual morph: conidiogenous cells and conidia. **(D–J)** Morphology of *Leptographium linjiangense* sp. nov (Taxon 4). **(D)** Cultures on 2% MEA 10 days after inoculation. **(E–G)** Leptographium-like asexual morph: conidiogenous cells and conidia. **(H–J)** Hyalorhinocladiella-like asexual morph: conidiogenous cells and conidia. **(K–N)** Morphology of *Leptographium qieshaoense* sp. nov (Taxon 5). **(K)** Cultures on 2% MEA 10 days after inoculation. **(L–N)** Leptographium-like asexual morph: conidiogenous cells and conidia. Scale bars: 20 μm **(E,G)**, 10 μm **(B,C,F,H–J,L–N)**.

Description: Sexual form: unknown. Asexual forms: Hyalorhinocladiella-like. Conidiophores arise directly from aerial hyphae, simple or loosely branched, macronematous or semi-macronematous, mononematous, the ultimate branched, bearing conidiogenous cells. Conidiogenous cells are solitary, hyaline, think-walled, with rounded apex (3.22–) 7.77 – 19.15 (−28.43) × (0.7–) 0.82 – 1.24 (−1.7) μm. Conidia are hyaline, aseptate, 1-celled, smooth, oval to elliptical (2.36–) 2.73 – 3.41 (−3.94) × (0.66–) 0.73 – 1.35 (−2.95) μm (Figures 7B,C).

Culture characteristics: Colonies on 2% MEA at 25°C in the dark, reaching 24.8 mm in diameter in 10 days. The colonies had smooth margins and were hyaline to white in color. Mycelia were compact with sparse aerial mycelia. The optimal growth temperature was 30°C; no growth was observed at 5°C, and litter growth occurred at 35°C (Figure 7A).

Known hosts: *P. koraiensis*.

Known insect vectors: *T. pilifer*.

Known distribution: Jilin Province, China.

Additional specimens examined: China. Jilin Province, Linjiang City, adults of *T. pilifer* in *P. koraiensis*. Jun. 2020, H.M. Wang, CFCC57453 = CXY4032.

Notes: *Ceratocystiopsis changbaiensis* was only characterized by a Hyalorhinocladiella-like reproductive structure on 2% MEA. Based on the phylogenetic evidence and morphological features, our strains were identified as novel species. *Ceratocystiopsis changbaiensis* was phylogenetically close to *Ceratocystiopsis* sp. 2 (Figures 1, 2). Nevertheless, the vectors and substrates of the two species were different. *Ceratocystiopsis changbaiensis* was isolated from *T. pilifer* infesting *P. koraiensis* in China, and *Ceratocystiopsis* sp. 2 was isolated from *Ips subelongatus* infesting *Larix kaempferi* in Japan (Reid and Hausner, 2010; De Beer and Wingfield, 2013).

#### Leptographium linjiangense H.M. Wang and Q. Lu, sp. nov.

Mycobank MB843473

Etymology: The name refers to Linjiang City, where this fungus was collected.

Type: China. Jilin Province, Linjiang City, adults of *T*. *pilifer* in *P*. *koraiensis*. Jun. 2020, H.M. Wang, holotype CXY4044, culture ex-holotype CFCC57337 = CXY4044.

Description: Sexual forms: unknown. Asexual forms: Leptographium-like and Hyalorhinocladiella-like.

**Leptographium-like**: Conidiophores occur singly without rhizoid-like structures and arise from the superficial mycelium, erect, macronematous, mononematous, (44.39–) 79.14 – 194.62 (−281.51) μm long. The stipes are simple, constricted at septa, cylindrical, olivaceous at the base, 1–7 septate, (6.06–) 21.11 – 78.77 (−120.26) × (3.35–) 4.16 – 8.16 (−11.31) μm. Conidiogenous apparatus with 1–3 series of cylindrical branches. The primary branches are hyaline to pale olivaceous in color, smooth, cylindrical, 2–7 branch, (6.06–) 9.32 – 29.18 (−60.98) × (2.81–) 3.61 – 7.63 (−12.29) μm. Conidiogenous cells are discrete, cylindrical, (12.2–) 17 – 34.04 (−56) × (2.38–) 2.85 – 3.75 (−4.21) μm. Conidia are 1-celled, hyaline, aseptate, globose, and elliptical to drop with truncate bases (3.52–) 5.76 – 10.76 (−14.26) × (2.43–) 2.99 – 4.87 (−6.75) μm.

**Hyalorhinocladiella-like**: Conidiophores arise directly from aerial hyphae, reduced to conidiogenous cells, macronematous or semi-macronematous, and mononematous. Conidiogenous cells are solitary, simple, hyaline to pale brown in color, aseptate, thin-walled, and variable in length (13.44–) 19.06 – 54.52 (−90.96) × (1.78–) 2.37 – 3.71 (−4.2) μm. Conidia are hyaline, smooth, oval to elliptical with truncate bases, 1-celled, aseptate, (4.93–) 6.29 – 9.39 (−12) × (2.33–) 3.02 – 4.48 (−5.68) μm (Figures 7E–J).

Culture characteristics: Colonies were cultured on 2% MEA at 25°C in the dark, reaching 90 mm in diameter in 7 days. Hyphae were submerged with few aerials, hyaline at first, later becoming dark brown. The mycelia were superficial. The optimal growth temperature was 30°C; no growth was observed at 5°C, and little growth was seen at 35°C (Figure 7D).

Known hosts: *P. koraiensis*.

Known insect vectors: *T. pilifer*.

Known distribution: Jilin Province, China.

Additional specimens examined: China. Jilin Province, Linjiang City, adults of *T. pilifer* in *P. koraiensis*. Jun. 2020, H.M. Wang, CFCC57338 = CXY4045, CFCC57339 = CXY4046.

Notes: *Leptographium linjiangense* produced Leptographium-like and Hyalorhinocladiella-like asexual states *in vitro*. The species was phylogenetically closest to *L. shansheni* based on TUB2, TEF1-α, and combined (ITS2 + TUB2 + TEF1-α) trees (Figure 4 and Supplementary Figures S3, S4). *Leptographium linjiangense* produced two asexual states, whereas *L. shansheni* only produced Leptographium-like asexual state. The stipes and primary branches of the Leptographium-like state between *L. linjiangense* and *L. shansheni* differed, as the former is longer than the latter (Stipes: 1–7 septate, 44.39–281.51 μm *vs*. 1 septate, 9–23 μm. Primary branches: primarily 6.06–60.98 μm *vs*. mostly 6.5–27.5 μm; Chang et al., 2019). The optimal growth temperatures for *L. linjiangense* and *L. shansheni* were 30 and 25°C, respectively, on 2% MEA. *Leptographium linjiangense* exhibited little growth at 35°C, whereas *L. shansheni* did not grow at 35°C. Furthermore, *L. linjiangense* and *L. shansheni* were associated with *T. pilifer* and *I. typographus*, respectively.

#### Leptographium qieshaoense H.M. Wang and Q. Lu, sp. nov.

Mycobank MB843474

Etymology: The name refers to the Chinese word for pine shoot beetles, “qieshao”.

Type: China. Jilin Province, Linjiang City, adults of *T. pilifer* in *P. koraiensis*. Jun. 2020, H.M. Wang, holotype CXY4038, culture ex-holotype CFCC57328 = CXY4038.

Description: Sexual form: unknown. Asexual forms: Leptographium-like. Conidiophores occur singly without rhizoid-like structures and arise from the superficial mycelium, erect, macronematous, mononematous, (43.68–) 52.77 – 97.43 (−166.63) μm long. The stipes are simple, constricted at septa, cylindrical, and pale olivaceous in color at the base, 1–6 septate, (3.89–) 17.44 – 43.68 (−67.33) × (1.94–) 2.69 – 4.53 (−6.73) μm. Conidiogenous apparatus with 2–3 series of cylindrical branches; primary branches hyaline to pale olivaceous, smooth, cylindrical, 2–3 branches, (7.44–) 9.96 – 14.7 (−19.41) × (1.97–) 2.28 – 3.34 (−4.05) μm. Conidiogenous cells are discrete, 1–3 per branch, cylindrical, (9.45–) 10.86 – 17.5 (−23.9) × (0.87–) 1.43 – 2.15 (−2.42) μm. Conidia are 1-celled, hyaline, cylindrical to obovoid with truncate bases, aseptate (3.61–) 4.46 – 6.24 (−7.51) × (1.63–) 1.87 – 2.87 (−3.55) μm (Figures 7L–N).

Culture characteristics: Colonies on 2% MEA at 25°C in the dark, reaching 90 mm in diameter in 7 days, with radial colony margins, initially hyaline, later becoming greenish-olivaceous to olivaceous, with abundant aerial mycelia. The optimal growth temperature was 30°C; no growth was observed at 5°C, and little growth was seen at 35°C (Figure 7K).

Known hosts: *P. koraiensis*.

Known insect vectors: *T. pilifer*.

Known distribution: Jilin Province, China.

Additional specimens examined: China. Jilin Province, Linjiang City, *T. pilifer* adults found in *P. koraiensis*. Jun. 2020, H.M. Wang, CFCC57327 = CXY4039, CFCC57345 = CXY4041, CFCC57344 = CXY4043; China. Jilin Province, Tonghua City, adults of *T. pilifer* in *P. koraiensis*. Jul. 2020, H.M. Wang, CFCC57343 = CXY4040, CFCC57342 = CXY4042.

Notes: *Leptographium qieshaoense* had a single asexual state and it is Leptographium-like. It was closely related to *L. koreanum* and *L. pinicola* based on ITS2-LSU and combined (ITS2 + TUB2 + TEF1-α) phylogenetic analyses (Figures 4, 5). However, *L. qieshaoense* and *L. koreanum* differed in their conidia, conidiogenous cells, and stipes of asexual states. The conidiogenous cells of the former were longer than those of the latter (primarily 9.45–23.9 μm *vs*. mostly 6–11 μm). In contrast, the stipes of *L. qieshaoense* were shorter than those *L. koreanum* (3.89–67.33 *vs*. 35–227 μm), and the conidia size range of *L. qieshaoense* was shorter than that of *L. koreanum* (3.61–7.51 μm *vs*. 3–10 μm; Kim et al., 2005). The conidiogenous cells of *L. qieshaoense* and *L. pinicola* were different. The former was shorter than the latter (primarily 9.45–23.9 μm *vs*. mostly 11–48 μm; Jacobs et al., 2005). Moreover, the optimal temperatures of *L. qieshaoense, L. koreanum*, and *L. pinicola* were different, 30, 25, and 25 °C, respectively. The insect vectors, hosts, and collection sites for the three species were different. *Leptographium qieshaoense* is associated with *T. pilifer* infesting *P. koraiensis* in China; *L. koreanum* is associated with *T. piniperda, Hylurgops interstitialis, Hylastes paralleus*, and *H. plumbeus* infesting *Pinus densiflora* and *P. koraiensis* in Korea and Japan; *L. pinicola* is associated with *Hylastes* infesting *P. resinosas* and *P. densiflora* in Canada and Japan (Jacobs et al., 2005; Kim et al., 2005; Masuya et al., 2005).

#### Ophiostoma piliferi H.M. Wang and Q. Lu, sp. nov.

Mycobank MB843475

Type: China. Jilin Province, Linjiang City, adults of *T. pilifer* in *P. koraiensis*. Jun. 2020, H.M. Wang, holotype CXY4048, culture ex-holotype CFCC57370 = CXY4048.

Etymology: The name refers to the bark beetle vector of *T. pilifer*.

Description: Sexual form: unknown. Asexual forms: Hyalorhinocladiella-like.

**Figure 8:**
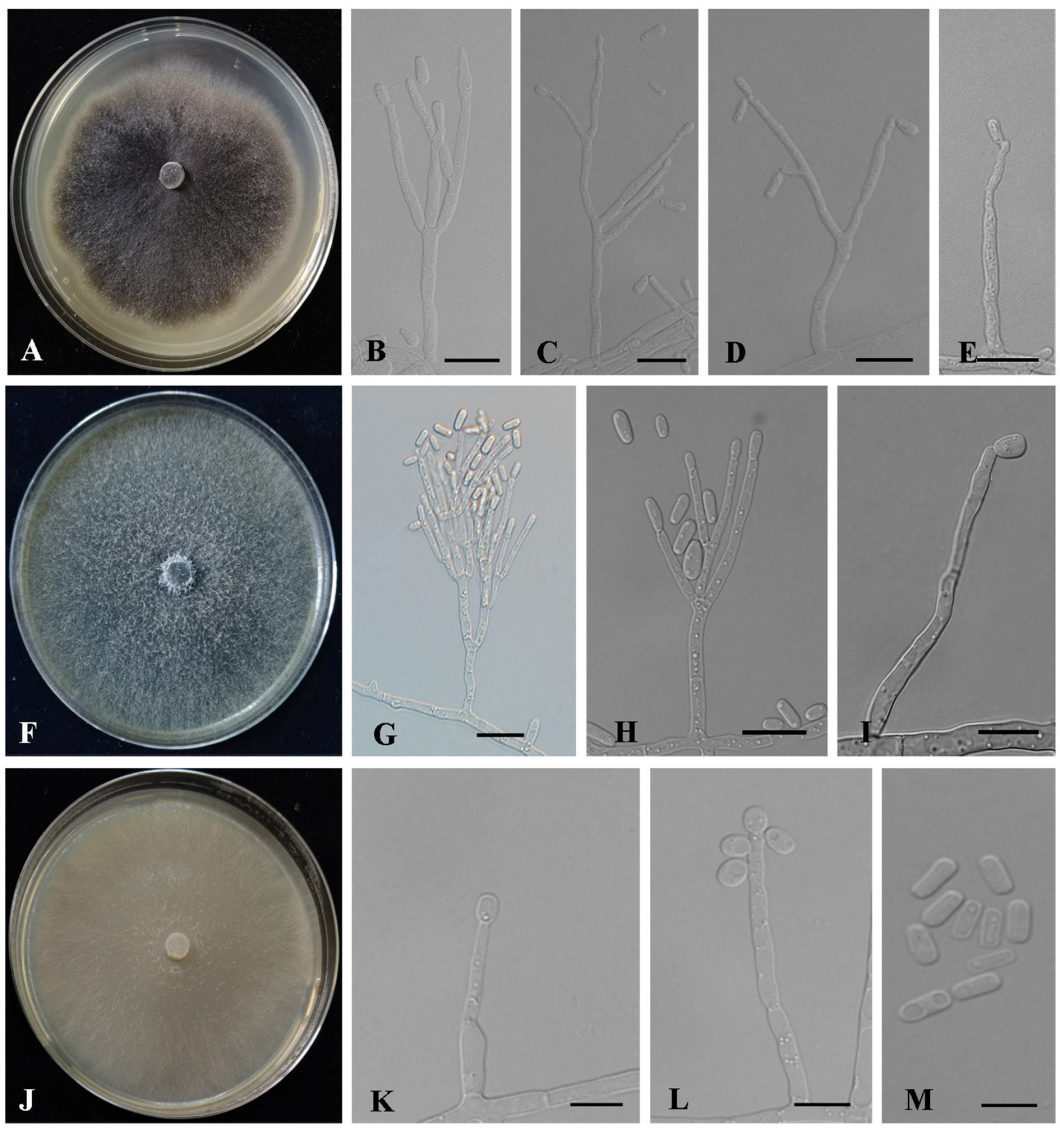
Morphology characteristics of *Ophiostoma* new species. **(A–E)** Morphology of *Ophiostoma piliferi* sp. nov (Taxon 7). **(A)** Cultures on 2% MEA 10 days after inoculation. **(B–E)** Hyalorhinocladiella-like asexual morph: conidiogenous cells and conidia. **(F–I)** Morphology of *Ophiostoma tonghuaense* sp. nov (Taxon 8). **(F)** Cultures on 2% MEA 10 days after inoculation. **(G,H)** Leptographium-like asexual morph: conidiogenous cells and conidia. **(I)** Hyalorhinocladiella-like asexual morph: conidiogenous cells and conidia. **(J–M)** Morphology of *Ophiostoma tonghuaense* sp. nov (Taxon 9). **(J)** Cultures on 2% MEA 10 days after inoculation. **(K–M)** Hyalorhinocladiella-like asexual morph: conidiogenous cells and conidia. Scale bars: 20 μm **(G)**; 10 μm **(B–E,H–I,K–M)**.

**Hyalorhinocladiella-like**: Conidiophores semi-macronematous, mononematous, arise directly from aerial hyphae, simple or loosely branched, the ultimate branches bearing conidiogenous cells. Conidiogenous cells are hyaline, smooth, thin-walled, aseptate (5.44–) 12.19 – 30.73 (−55.74) × (1. 12–) 1.41 – 2.08 (−3.13) μm. Conidia are hyaline, smooth, 1-celled, clavate to elliptical with pointed bases and rounded apices, aseptate, (2.81–) 4.07 – 5.99 (−8.76) × (1.2–) 1.62 – 2.38 (−3.41) μm (Figures 8B–E).

Culture characteristics: Colonies on 2% MEA at 25°C in the dark, reaching 65 mm in diameter in 10 days, with white hyphae appressed to flocculose, dark brown to black, and anomalous mycelium margins. The optimal growth temperature was 30°C. No growth was observed at 5°C, and little growth was seen at 35°C (Figure 8A).

Known hosts: *P. koraiensis*.

Known insect vectors: *T. pilifer*.

Known distribution: Jilin Province, China.

Additional specimens examined: China. Jilin Province, Linjiang City, adults of *T. pilifer* in *P. koraiensis*. Jun. 2020, H.M. Wang, CFCC57410 = CXY4047, CFCC57371 = CXY4049; China. Jilin Province, Tonghua City, adults of *T. pilifer* in *P. koraiensis*. Jul. 2020, H.M. Wang, CFCC57377 = CXY4050, CFCC57376 = CXY4051, CFCC57372 = CXY4052.

Notes: *Ophiostoma piliferi* produced Hyalorhinocladiella-like asexual states in 2% MEA. ITS and combined (TUB2 + TEF1-α) phylogenetic analyses showed that *O. piliferi* is closely related to *O. shennongensis, O. tonghuaense*, and *Ophiostoma* sp. 2 (Figures 2, 6). (cf. below for *O. tonghuaense*).

#### Ophiostoma tonghuaense H.M. Wang and Q. Lu, sp. nov.

Mycobank MB843477

Type: China, Jilin Province, Tonghua City, adults of *T. pilifer* in *P. koraiensis*. Jun. 2020, H.M. Wang, holotype CXY4053, culture ex-holotype CFCC57374 = CXY4055.

Etymology: The name refers to Tonghua City, where this fungus was collected.

Description: Sexual form: unknown. Asexual forms: Leptographium-like and Hyalorhinocladiella-like.

**Leptographium-like**: Conidiophores occurring singly arise from the superficial mycelium, without rhizoid-like structures, macronematous, mononematous, (28.71–) 42.39 – 93.95 (−142.14) μm long. The stipes are simple, hyaline, not constricted at septa, cylindrical, 1–4 septate, (7.79–) 10.73 – 39.51 (−75.31) × (1.98–) 2.48 – 3.98 (−5.55) μm. Conidiogenous apparatus with 2–3 series of cylindrical branches. The primary branches are cylindrical, hyaline, smooth, 2–3 branches, (6.12–) 9.1 – 18.86 (−27.49) × (1.63–) 2 – 3.36 (−4.84) μm. Conidiogenous cells are discrete, hyaline, smooth, cylindrical, (9.41–) 12.09 – 24.79 (−45.62) × (1.55–) 1.79 – 2.65 (−3.23) μm. Conidia are 1-celled, hyaline, clavate to elliptical, aseptate, (9.41–) 12.09 – 24.79 (−45.62) × (1.55–) 1.79 – 2.65 (−3.23) μm.

**Hyalorhinocladiella-like**: Conidiophores arise directly from aerial hyphae, solitary or loosely branched, macronematous or semi-macronematous, mononematous, ultimate branched bearing conidiogenous cells. Conidiogenous cells are simple, hyaline to pale brown, aseptate or sparsely septate, thin-walled, and variable in length (12.43–) 13.66 – 56.86 (−81.78) × (1.43–) 1.68 – 3.1 (−3.98) μm. Conidia are hyaline, 1-celled, smooth, aseptate, oval to elliptical with obtuse ends, (2.48–) 3.93 – 6.21 (−7.71) × (1.25–) 1.58 – 2.6 (−3.69) μm (Figures 8G–I).

Culture characteristics: Colonies on 2% MEA at 25°C in the dark, reaching 90 mm in diameter in 5 days. The colony margin was anomalous, with abundant aerial mycelia, flocculose, and hyaline initially, becoming greenish-olivaceous to olivaceous. The optimal growth temperature was 30°C. No growth was observed at 5°C, and litter growth was achieved at 35°C (Figure 8F).

Known hosts: *P. koraiensis*.

Known insect vectors: *T. pilifer*.

Known distribution: Jilin Province, China.

Additional specimens examined: China. Jilin Province, Linjiang City, adults of *T. pilifer* in *P. koraiensis*. Jun. 2020, H.M. Wang, CFCC57378 = CXY4053, CFCC57375 = CXY4054, CFCC57384 = CXY4056.

Notes: *Ophiostoma tonghuaense* produced Leptographium-like and Hyalorhinocladiella-like asexual states in 2% MEA. ITS and combined (TUB2 + TEF1-α) phylogenetic analyses showed that *O. tonghuaense* is grouped with *Ophiostoma* sp. B (Chang et al., 2017) and is closely related to *O. piliferi* and *O. shennongense* (Figures 2, 6). *Ophiostoma* sp. B was obtained from only one strain isolated from the gallery of an unknown beetle infesting *Pinus semaonensis*; this species has not been formally described in previous studies (Chang et al., 2017). Therefore, this did not influence the formal description of *O. tonghuaense*.

*Ophiostoma tonghuaense, O. shennongense*, and *O. piliferi* differed in their asexual states. *Ophiostoma tonghuaense* produces Leptographium-like and Hyalorhinocladiella-like asexual states; *O. shennongense* and *O. piliferi* produces a single Hyalorhinocladiella-like asexual state (Figure 8; Wang et al., 2022). Among the three species, the growth rate of *O. tonghuaense* at 25°C was the fastest (90 mm in diameter in 5 days), followed by *O. shennongense* (70 mm in diameter in 8 days) and *O. piliferi* (65 mm in diameter in 10 days).

The culture color of *O. tonghuaense, O. piliferi*, and *O. shennongense* on 2% MEA at 25°C was greenish-olivaceous to olivaceous, dark brown to black, and pale olivaceous, respectively. Although the geographic distribution of these three *Ophiostoma* species overlaps, their hosts and vectors are different. *Ophiostoma tonghuaense* and *O. piliferi* are associated with *T. pilifer* infesting *P. koraiensis*, whereas *O. shennongense* is associated with *Dendroctonus armandi* infesting *P. armandii*.

#### Ophiostoma yaluense H.M. Wang and Q. Lu, sp. nov.

Mycobank MB843479

Type: China, Jilin Province, Tonghua City, adults of *T. pilifer* in *P. koraiensis*. Jun. 2020, H.M. Wang, holotype CXY4059, culture ex-holotype CFCC57382 = CXY4059.

Etymology: The name refers to the Yalujiang river where this species was collected.

Description: Sexual form: unknown. Asexual forms: Hyalorhinocladiella-like. Conidiophores are solitary branched, arise directly from aerial hyphae, and are reduced to conidiogenous cells, macronematous or semi-macronematous, mononematous, simple, hyaline, aseptate, sparsely septate, thin-walled, and variable in length (7.04–) (16.8) – (62.52) (−98.4) × (1.37–) (1.85) – (2.57) (−2.89) μm. Conidia are aseptate, hyaline, smooth, 1-celled, globose, oval to elliptical, with truncate bases (2.7–) (3.64) – (4.8) (−5.58) × (1.5–) (1.82) – (2.72) (−3.35) μm (Figures 8K–M).

Culture characteristics: Colonies cultured on 2% MEA at 25°C in the dark reached 90 mm in diameter in 9 days. The colonies had smooth margins and were superficial and hyaline in color. The optimal growth temperature was 25°C, and slow growth was observed at 5 and 35°C (Figure 8J).

Known hosts: *P. koraiensis*.

Known insect vectors: *T. pilifer*.

Known distribution: Jilin Province, China.

Additional specimens examined: China. Jilin Province, Linjiang City, adults of *T. pilifer* in *P. koraiensis*. Jun. 2020, H.M. Wang, CFCC57381 = CXY4057, CFCC57380 = CXY4058.

Notes: *Ophiostoma yaluense* was found only in an asexual, Hyalorhinocladiella-like state. Phylogenetic analysis of the ITS and combined (TUB2 + TEF1-α) regions revealed that *O. yaluense* forms a separate lineage with high node support values (Figures 2, 6). Moreover, this species is phylogenetically closely related to *O. japonicum* based on TUB2 + TEF1-α phylogenetic data; however, the asexual of the two species were different. *Ophiostoma japonicum* produced Graphilbum-like asexual states (Yamaoka et al., 1997). In addition, vectors and hosts of both species were different. *Ophiostoma yaluense* was associated with *T. pilifer* infesting *P. koraiensis* and *O. japonicum* was associated with *I. typographus japonicus infesting P. jezoensis* (Japan) and *P. koraiensis* (China).

## Discussion

Herein, a total of 315 ophiostomatalean fungal strains representing nine taxa were isolated primarily from the adults of *T. pilifer* and secondarily from their galleries found in infested *P. koraiensis* trees in northeastern China. The results of this study revealed six new species (*Cop. changbaiensis, L. qieshaoense, L. linjiangense, O. piliferi, O. tonghuaense*, and *O. yaluense*), two known species (*Gra. interstitiale* and *O. fuscum*), and one undefined species (*Ceratocystiopsis* sp. 1). *Graphilbum interstitiale* and *O. fuscum* have not been previously reported in China. In this study, although the six novel species belonged to three different genera, five of them exhibited optimal growth at 30°C, no growth at 0.5°C, and limited growth at 35°C. Meanwhile, the optimal growth of *O*. *yaluense* was recorded at 25°C. In addition, we determined the taxonomic status of *T. pilifer* based on 28S rDNA phylogenetic analysis (Figure 1), which provides a molecular basis for subsequent studies on the classification of *Tomicus* species.

The new species identified in this study can be easily distinguished from other known species by integrating phylogenetic analysis, and examining their physiological and morphological characteristics. *Tomicus pilifer* and ophiostomatalean fungi obtained in this study form a new association of beetle-fungal. Therefore, the following discussion focuses on the association between fungi diversity and beetles.

*Ophiostoma piliferi* was the most frequently isolated ophiostomatalean fungus associated with *T. pilifer* in our study (124 out of the 315 strains), accounting for 39.37% (Table 2). *Ophiostoma piliferi* and two other new species, *O. tonghuaense* and *O. yaluense* were classified in the *O. clavatum* complex (Figure 2). Noteworthily, *O. tonghuaense* and *O. yaluense* included 19 and 6 strains, accounting for 6.03% and 1.9% of the total ophiostomatalean fungal strains, respectively.

The fungal diversity of the *O. clavatum* complex was high, and a total of 22 taxa have been documented, including the three new species obtained in this study (Linnakoski et al., 2016; De beer et al., 2022; Wang et al., 2022; Figure 6 and Supplementary Figures S5, S6). All species of the *O. clavatum* complex are associated with bark beetles infesting pine or spruce. In addition, multiple new ophiostomatalean fungi of the *O. clavatum* complex have often been simultaneously isolated from identical bark beetles or hosts. For example, *O. jiamusiensis, O. songshui, O. ainoae*, and *O. brunneolum* are associated with *I. typographus* (Chang et al., 2017). *Ophiostoma hongxingense, O. subelongati*, and *O. peniculi* were obtained from *I. subelongatus* infesting *L. gmelinii* (Wang et al., 2020). *Ophiostoma brevipilosi* was the first species in the *O. clavatum* complex associated with *Tomicus* (Chang et al., 2017). Subsequently, this study isolated and identified three new species of *O. clavatum* complex associated with *T. pilifer*.

In our study, the second most abundant species was *L. qieshaoense* (113 out of the 315 strains), accounting for 35.87% (Table 2), which forms part of the *L. lundbergii* complex (Figure 4). In addition, *L. linjiangense* was grouped in this complex, with a total of 12 strains, accounting for 3.81%. *Leptographium lundbergii* is a species of *Leptographium* described in 1927 (Jacobs et al., 2005). The *L. lundbergii* complex was defined by Jacobs et al. (2005), and is currently composed of 17 taxa, including the two new species obtained in this study (Chang et al., 2019; Pan et al., 2020a,b; De beer et al., 2022; Wang et al., 2022; Figure 5 and Supplementary Figures S3, S4). All species documented in this complex were isolated from conifers (Jacobs et al., 2005; Linnakoski et al., 2012b; Chang et al., 2019).

Regarding the two newly recorded species, *Gra. interstitiale* was first isolated from *H*. *interstitialis* infesting *Pinus sylvestris* in the far east of Russia (Jankowiak et al., 2020), whereas *O. fuscum* was first obtained from *I. typographus* and *Pityogenes chalcographus* infesting *P. abies* in Russia (Linnakoski et al., 2010). In this study, 26 and 9 strains of *Gra. interstitiale* and *O. fuscum* were collected, accounting for 8.25% and 3.81% of the strains, respectively. Despite the small number of strains, *Gra. interstitiale* was the third most abundant ophiostomatalean fungus collected (Table 2). These two newly recorded species were first reported to be associated with *Tomicus*. Many new and newly recorded species were obtained in this study, indicating that a large number of ophiostomatalean fungi associated with *T. pilifer* are yet to be identified.

Thus far, 61 ophiostomatalean fungi (including the nine species obtained in this study) were reported to be associated with seven *Tomicus* species, among which *T. piniperda* had the highest diversity of ophiostomatalean fungi, followed by *T. minor, T. yunnanense, T. pilifer*, and *T. destruens*, whereas *T. brevipilosus* and *T. armandii* had the lowest diversity with only three species (Table 1 and Supplementary Table S1). This phenomenon may be explained by the wider distribution of *T. piniperda* and *T. minor* as compared with other *Tomicus* species, spanning various climatic regions and diverse hosts (Masuya et al., 1999a,b; Jacobs and Wingfield, 2001; Jacobs et al., 2003; Jankowiak, 2006; Jankowink, 2006; Linnakoski et al., 2010). In addition, the study on ophiostomatalean fungi associated *T. piniperda* and *T. minor* began earlier, and the samples analyzed were obtained from a larger area (Mathiesen-Kaarik, 1953; Masuya et al., 1999a,b). Therefore, the documented ophiostomatalean fungi associated *T. piniperda* and *T. minor* had the highest diversity among the seven beetles. In contrast, the other *Tomicus* species were found to only have a few associated ophiostomatalean fungi.

An increasing number of studies have shown that ophiostomatalean fungal symbiose with bark and ambrosia beetles (Biedermann and Vega, 2020). This study determined the diversity of ophiostomatalean fungi associated with *T. pilifer*-infested *P. koraiensis* tissues in northeastern China. Fungal classification is the basis for the research on fungal function. Therefore, our findings provide basic knowledge for understanding the relationship between bark beetles and ophiostomatalean fungi and aid in designing strategies for the management of *Tomicus*.

## Data availability statement

The datasets presented in this study can be found in online repositories. The names of the repository/repositories and accession number(s) can be found in the article/Supplementary material.

## Author contributions

HW designed the study, analyzed the data and contributed to experiment design. HW and FY collected the samples. HW and CL performed DNA extraction and PCR amplification. QL and HW wrote the manuscript. QL and D-HY reviewed and approved the final manuscript. All authors have read and agreed to the published version of the manuscript. All authors contributed to the article and approved the submitted version.

## Funding

This study was supported by the National Natural Science Foundation of China (Project No. 32071769).

## Conflict of interest

The authors declare that the research was conducted in the absence of any commercial or financial relationships that could be construed as a potential conflict of interest.

## Publisher's note

All claims expressed in this article are solely those of the authors and do not necessarily represent those of their affiliated organizations, or those of the publisher, the editors and the reviewers. Any product that may be evaluated in this article, or claim that may be made by its manufacturer, is not guaranteed or endorsed by the publisher.

## Supplementary material

Supplementary Table S1 Diversity of ophiostomatalean fungi associated with *Tomicus* spp.

Supplementary Table S2 Reference sequences which are involved in phylogenetic tree in this study.

Supplementary Figure S1 ML tree of *Graphilbum* generated from the TUB2 sequence data. Novel sequences obtained in this study are presented in bold typeface. The bold branches indicate posterior probability values >0.9. Bootstrap values of ML ≥ 70% are recorded at the nodes. T, ex-type strains. The final alignment of 501 positions, including gaps.

Supplementary Figure S2 ML tree of *Graphilbum* generated from the EF1-α sequence data. Novel sequences obtained in this study are presented in bold typeface. The bold branches indicate posterior probability values >0.9. Bootstrap values of ML ≥ 70% are recorded at the nodes. T, ex-type strains. The final alignment of 704 positions, including gaps.

Supplementary Figure S3 ML tree of *L. lundbergii* complex generated from the TUB2 (Taxon 4, 5) sequence data. Novel sequences obtained in this study are presented in bold typeface. The bold branches indicate posterior probability values >0.9. Bootstrap values of ML ≥ 70% are recorded at the nodes. T, ex-type strains. The final alignment of 369 positions, including gaps.

Supplementary Figure S4 ML tree of *L. lundbergii* complex generated from the EF1-α (Taxon 4, 5) sequence data. Novel sequences obtained in this study are presented in bold typeface. The bold branches indicate posterior probability values >0.9. Bootstrap values of ML ≥ 70% are recorded at the nodes. T, ex-type strains. The final alignment of 635 positions, including gaps.

Supplementary Figure S5 ML tree of *O. clavatum* complex generated from the TUB2 (Taxon 7, 8, 9) sequence data. Novel sequences obtained in this study are presented in bold typeface. The bold branches indicate posterior probability values >0.9. Bootstrap values of ML ≥ 70% are recorded at the nodes. T, ex-type strains. The final alignment of 388 positions, including gaps.

Supplementary Figure S6 ML tree of *O. clavatum* complex generated from the EF1-α (Taxon 7, 8, 9) sequence data. Novel sequences obtained in this study are presented in bold typeface. The bold branches indicate posterior probability values >0.9. Bootstrap values of ML ≥ 70% are recorded at the nodes. T, ex-type strains. The final alignment of 793 positions, including gaps.

Supplementary Figure S7 ML tree of *O. ips* complex generated from the TUB2 (Taxon 6) sequence data. Novel sequences obtained in this study are presented in bold typeface. The bold branches indicate posterior probability values >0.9. Bootstrap values of ML ≥ 70% are recorded at the nodes. T, ex-type strains. The final alignment of 414 positions, including gaps.

## References

[B1] Ayres M. P. Wilkens R. T. Ruel J. J. Lombardero M. J. Vallery E. (2000). Nitrogen budgets of phloem-feeding bark beetles with and without symbiotic fungi. Ecology 81, 2198–2210. 10.1890/0012-9658(2000)081[2198:NBOPFB]2.0.CO;2

[B2] Biedermann P. H. W. Vega F. E. (2020). Ecology and evolution of insect–fungus mutualisms. Annu. Rev. Entomol. 5, 431–455. 10.1146/annurev-ento-011019-024910 31610133

[B3] Bleiker K. P. Six D. L. (2007). Dietary benefits of fungal associates to an eruptive herbivore: potential implications of multiple associates on host population dynamics. Environ. Entomol. 36, 1384–1396. 10.1093/ee/36.6.1384 18284766

[B4] Cale J. A. Ding R. Wang F. Rajabzadeh R. Erbilgin N. (2019). Ophiostomatoid fungi can emit the bark beetle pheromone verbenone and other semiochemicals in media amended with various pine chemicals and beetle-released compounds. Fungal Ecol. 39, 285–295. 10.1016/j.funeco.2019.01.003

[B5] Chang R. Duong T. A. Taerum S. J. Wingfield M. J. Zhou X. De Beer Z. W. (2017). Ophiostomatoid fungi associated with conifer-infesting beetles and their phoretic mites in Yunnan, China. MycoKeys 28, 19–64. 10.3897/mycokeys.28.21758 29559821 PMC5804140

[B6] Chang R. Duong T. A. Taerum S. J. Wingfield M. J. Zhou X. Yin M.. (2019). Ophiostomatoid fungi associated with the spruce bark beetle *Ips typographus*, including 11 new species from China. Persoonia Mol. Phylo. Evol. Fungi 42, 50–74. 10.3767/persoonia.2019.42.03 31551614 PMC6712535

[B7] De beer Z. W. Procter M. Wingfield M. J. Marincowitz S. Duong T. A. (2022). Generic boundaries in the Ophiostomatales reconsidered and revised. Stud. Mycol. 101, 57–120. 10.3114/sim.2022.101.02 36059894 PMC9365045

[B8] De Beer Z. W. Wingfield M. J. (2013). Emerging lineages in the Ophiostomatales, in The Ophiostomatoid Fungi: Expanding Frontiers, eds De Beer Z. W. Wingfield M. J. (Utrecht: CBS), 21–46.

[B9] DiGuistini S. Wang Y. Liao N. Y. Taylor G. Tanguay P. Feau N.. (2011). Genome and transcriptome analyses of the mountain pine beetle-fungal symbiont *Grosmannia clavigera*, a lodgepole pine pathogen. PNAS 108, 2504–2509. 10.1073/pnas.1011289108 21262841 PMC3038703

[B10] Duan Y. Kerdelhue C. Ye H. Lieutier F. (2004). Genetic study of the forest pest Tomicus piniperda (Col., Scolytinae) in Yunnan Province (China) compared to Europe: new insights for the systematics and evolution of the genus *Tomicus*. Heredity 93, 416–422. 10.1038/sj.hdy.6800518 15280894

[B11] Gallego D. Galián J. (2001). The internal transcribed spacers (ITS1 and ITS2) of the rDNA differentiates the bark beetle forest pests *Tomicus destruens* and *T. piniperda*. Insect Mol. Biol. 10, 415–420. 10.1046/j.0962-1075.2001.00279.x 11881805

[B12] Gardes M. Bruns T. D. (1993). ITS primers with enhanced specificity for basidiomycetes-application to the identification of mycorrhizae and rusts. Mol. Ecol. 2, 113–118. 10.1111/j.1365-294X.1993.tb00005.x 8180733

[B13] Glass N. L. Donaldson G. C. (1995). Development of primer sets designed for use with the PCR to amplify conserved genes from filamentous ascomycetes. Appl. Environ. Microb. 61, 1323–1330. 10.1128/aem.61.4.1323-1330.1995 7747954 PMC167388

[B14] Grégoire J. C. Raffa K. F. Lindgren B. S. (2015). Economics and politics of bark beetles, in Bark Beetles: Biology and Ecology of Native and Invasive Species, eds Vega F. E. Hofstetter R. W. (San Diego: Academic Press), 585–613. 10.1016/B978-0-12-417156-5.00015-0

[B15] Jacobs K. Bergdahl D. R. Wingfield M. J. Halik S. Seifert K. A. Bright D. E.. (2004). *Leptographium wingfieldii* introduced into North America and found associated with exotic *Tomicus piniperda* and native bark beetles. Mycol. Res. 108, 411–418. 10.1017/S0953756204009748 15209281

[B16] Jacobs K. Kirisits T. Wingfield M. J. (2003). Taxonomic re-evaluation of three related species of Graphium, based on morphology, ecology and phylogeny. Mycologia 95, 714–727. 10.1080/15572536.2004.11833075 21148980

[B17] Jacobs K. Solheim H. Wingfield B. D. Wingfield M. J. (2005). Taxonomic re-evaluation of *Leptographium lundbergii* based on DNA sequence comparisons and morphology. Mycol. Res. 109(Pt 10), 1149–1161. 10.1017/S0953756205003618 16279409

[B18] Jacobs K. Wingfield M. J. (2001). Leptographium Species: Tree Pathogens, Insect Associates, and Agents of Blue Stain. St. Paul: American Phytopathological Society Press.

[B19] Jankowiak R. (2006). Fungi associated with *Tomicus piniperda* in Poland and assessment of their virulence using Scots pine seedlings. Ann. For. Sci. 63, 801–808. 10.1051/forest:2006063

[B20] Jankowiak R. Solheim H. Bilański P. Marincowitz S. Wingfield M. J. (2020). Seven new species of *Graphilbum* from conifers in Norway, Poland, and Russia. Mycologia 112, 1240–1262. 10.1080/00275514.2020.1778375 32634330

[B21] Jankowink R. (2006). Fungi associated with *Tomicus minor* on *Pinus sylvestris* in Poland and their succession into the sapwood of beetle-infested windblown trees. Can. J. For. Res. 38, 2579–2588. 10.1139/X08-101

[B22] Kerdelhué C. Roux-Morabito G. Forichon J. Chambon J. M. Robert E. Lieutier F. (2002). Population genetic structure of *Tomicus piniperda* L. (Curculionidae: Scolytinae) on different pine species and validation of *T. destruens* (Woll.). Mol. Ecol. 11, 483–494. 10.1046/j.0962-1083.2002.01460.x 11918783

[B23] Kim J. J. Lim Y. W. Breuil C. Wingfield M. J. Zhou X. D. Kim G. H. (2005). A new *Leptographium* species associated with *Tomicus piniperda* infesting pine logs in Korea. Mycol. Res. 109, 275–284. 10.1017/S0953756204002060 15912944

[B24] Kirisits T. (2004). Fungal associates of European bark beetles with special emphasis on the ophiostomatoid fungi, in Bark and Wood Boring Insects in Living Trees in Europe, A Synthesis, eds Lieutier F. Day K. R. Battisti A. Grégoire J. C. Evans H. F. (The Netherlands: Kluwer Academic Publishers), 181–235. 10.1007/978-1-4020-2241-8_10

[B25] Kirkendall L. R. Faccoli M. Ye H. (2008). Description of the Yunnan shoot borer, *Tomicus yunnanensis* Kirkendall and Faccoli sp. n. (Curculionidae, Scolytinae), an unusually aggressive pine shoot beetle from southern China, with a key to the species of *Tomicus*. Zootaxa 1819, 25–39. 10.11646/zootaxa.1819.1.2

[B26] Kohlmayr B. Riegler M. Wegensteiner R. Stauffer C. (2002). Morphological and genetic identification of the three pine pests of the genus *Tomicus* (Coleoptera, Scolytidae) in Europe. Agr. For. Entomol. 4, 151–157. 10.1046/j.1461-9563.2002.00139.x

[B27] Lee S. Kim J. J. Breuil C. (2005). Leptographium longiclavatum sp. nov., a new species associated with the mountain pine beetle, *Dendroctonus ponderosae*. Mycol. Res. 109, 1162–1170. 10.1017/S0953756205003588 16279410

[B28] Lee S. Kim J. J. Breuil C. (2006). Diversity of fungi associated with mountain pine beetle, *Dendroctonus ponderosae*, and infested lodgepole pines in British Columbia. Fungal Diver. 22, 91–105.

[B29] Li X. Zhang Z. Wang H. B. Wu W. Cao P. Zhang P. Y. (2010). *Tomicus armandii* Li and Zhang (Curculionidae, Scolytinae), a new species from China. Zootaxa 2572, 57–64. 10.11646/zootaxa.2572.1.4

[B30] Liao Z. Y. Ye H. (2004). Action mechanisms of phytotoxin produced by *Leptographium yunnanense* associated with *Tomicus*. For. Pest Dis. 23, 20–22.

[B31] Lieutier F. Langstrom B. Faccoli M. (2015). The genus *Tomicus*, in Bark Beetles: Biology and Ecology of Native and Invasive Species, eds Vega F. E. Hofstetter R. W. (Amsterdam: Elsevier), 371–426. 10.1016/B978-0-12-417156-5.00010-1

[B32] Lieutier F. Yart A. Salle A. (2009). Stimulation of tree defenses by Ophiostomatoid fungi can explain attack success of bark beetles on conifers. Ann. For. Sci. 66, 801–822. 10.1051/forest/2009066

[B33] Linnakoski R. De Beer Z. W. Ahtiainen J. Sidorov E. Niemelä P. Pappinen A.. (2010). *Ophiostoma* spp. associated with pine-and spruce-infesting bark beetles in Finland and Russia. Persoonia Mol. Phylo. Evolut. Fungi 25, 72–93. 10.3767/003158510X550845 21339968 PMC3028507

[B34] Linnakoski R. de Beer Z. W. Duong T. A. Niemelä P. Pappinen A. Wingfield M. J. (2012b). *Grosmannia* and *Leptographium* spp. associated with conifer-infesting bark beetles in Finland and Russia, including *Leptographium taigense* sp. nov. Antonie van Leeuwenhoek 102, 375–399. 10.1007/s10482-012-9747-6 22580615

[B35] Linnakoski R. de Beer Z. W. Niemelä P. Wingfield M. J. (2012a). Associations of conifer-infesting bark beetles and fungi in fennoscandia. Insects 3, 200–227. 10.3390/insects3010200 26467956 PMC4553624

[B36] Linnakoski R. Jankowiak R. Villari C. Kirisits T. Solheim H. De Beer Z. W.. (2016). The *Ophiostoma clavatum* species complex: a newly defined group in the Ophiostomatales including three novel taxa. Antonie van Leeuwenhoek 109, 987–1018. 10.1007/s10482-016-0700-y 27142088

[B37] Lu M. Wingfield M. J. Gillette N. Sun J. H. (2011). Do novel genotypes drive the success of an invasive bark beetle-fungus complex? Implications for potential reinvasion. Ecology 92, 2013–2019. 10.1890/11-0687.1 22164824

[B38] Masuya H. Kaneko S. Yamaoka Y. Osawa M. (1999b). Comparisons of ophiostomatoid fungi associated with *Tomicus piniperda* and *T. minor* in Japanese red pine. J. For. Res. 4, 131–135.

[B39] Masuya H. Kaneko S. Yamaura Y. Yamaoka Y. (1999a). Ophiostomatoid fungi isolated from Japanese red pine and their relationships with bark beetles. Mycoscience 50, 212–223.

[B40] Masuya H. Kim J. J. Wingfield M. J. Yamaoka Y. Kaneko S. Breuil C.. (2005). Discovery and description of a teleomorph for *Leptographium koreanum*. Mycotaxon 94, 159–173.

[B41] Mathiesen-Kaarik A. (1953). Eine Ubersicht uber die gewohnlichsten mit Borkenkafern assoziierten Blauepilze in Schweden und einige fur Schweden neue Blauepilze. Meddelanden fran Statens Skogforskningsinstitut 43, 1–74.

[B42] Pan Y. Lu J. Zhou X. D. Chen P. Zhang H. Ye H. (2020a). Leptographium wushanense sp. nov., associated with *Tomicus armandii* on *Pinus armandii* in Southwestern China. Mycoscience 61, 43–48. 10.1016/j.myc.2018.10.003

[B43] Pan Y. Lu J. Zhou X. D. Yu Z. F. Chen P. Wang J.. (2020b). Two new species of Leptographium associated with Tomicus spp. infesting *Pinus* spp. in Southwestern China. Int. J. Syst. Evolut. Microbiol. 70, 4798–4807. 10.1099/ijsem.0.004349 32783804

[B44] Pan Y. Zhao T. Krokene P. Yu Z. F. Qiao J. Lu M.. (2018). Bark beetle-associated blue-stain fungi increase antioxidant enzyme activities and monoterpene concentrations in *Pinus yunnanensis*. Front. Plant Sci. 9, 1731. 10.3389/fpls.2018.01731 30559751 PMC6284243

[B45] Raffa K. F. Aukema B. H. Bentz B. J. Carroll A. L. Hicke J. A. Turner M. G.. (2008). Cross-scale drivers of natural disturbances prone to anthropogenic amplification: the dynamics of bark beetle eruptions. Bioscience 58, 501–517. 10.1641/B580607

[B46] Rayner R. W. (1970). A Mycological Colour Chart. London: CMI and British Mycological Society.

[B47] Reid J. Hausner G. (2010). The epitypification of *Ophiostoma minutum*, now *Ceratocystiopsis minuta*. Mycotaxon 113, 463–474. 10.5248/113.463

[B48] Ronquist F. Huelsenbeck J. P. (2003). MrBayes 3: Bayesian phylogenetic inference under mixed models. Bioinformatics 19, 1572–1574. 10.1093/bioinformatics/btg180 12912839

[B49] Six D. L. Wingfield M. J. (2011). The role of phytopathogenicity in bark beetle-fungus symbioses: a challenge to the classic paradigm. Ann. Rev. Entomol. 56, 255–272. 10.1146/annurev-ento-120709-144839 20822444

[B50] Stamatakis A. (2006). RAxML-VI-HPC: maximum likelihood-based phylogenetic analyses with thousands of taxa and mixed models. Bioinformatics 22, 2688–2690. 10.1093/bioinformatics/btl446 16928733

[B51] Wang H. Wang T. Liu Y. Zeng F. Zhang H. Lu Q.. (2022). Diversity of Ophiostomatoid fungi associated with *Dendroctonus armandi* infesting *Pinus armandii* in Western China. J. Fungi 8, 214. 10.3390/jof8030214 35330216 PMC8951329

[B52] Wang H. M. Wang Z. Liu F. Wu C. X. Zhang S. F. Kong X. B.. (2019). Differential patterns of ophiostomatoid fungal communities associated with three sympatric *Tomicus* species infesting pines in south-western China, with a description of four new species. MycoKeys 50, 93–133. 10.3897/mycokeys.50.32653 31043857 PMC6477840

[B53] Wang Y. C. Zhao B. Wang T. K. Li C. Ma L. Li G.. (2000). Study on occurring regulation and control method of *Tomicus pilifer*. Jilin For. Sci. Technol. 29, 10–11. 10.16115/j.cnki.issn.1005-7129.2000.05.004

[B54] Wang Z. Liu Y. Wang H. Meng X. Liu X. Decock C.. (2020). Ophiostomatoid fungi associated with *Ips subelongatus*, including eight new species from northeastern China. IMA Fungus 11, 1–29. 10.1186/s43008-019-0025-3 32617255 PMC7325231

[B55] White T. J. Bruns T. Lee S. Taylor J. (1990). Amplification and direct sequencing of fungal ribosomal RNA genes for phylogenetics, in PCR Protocols: A Guide to Methods and Applications, eds Gelfand I. M. A Sninsky D. H. White T. J. (San Diego: Academic Press), 18315–322.

[B56] Wingfield M. J. Eifert K. A. Webber J. F. (1993). Ceratocystis and Ophiostoma: Taxonomy, Ecology, and Pathogenicity. St. Paul, Minnesota: American Phytopathological Society, APS Press.

[B57] Wingfield M. J. Garnas J. R. Hajek A. Hurley B. P. de Beer Z. W. Taerum S. J. (2016). Novel and co-evolved associations between insects and microorganisms as drivers of forest pestilence. Biol. Invas. 18, 1045–1056. 10.1007/s10530-016-1084-7

[B58] Wingfield M. J. Slippers B. Wingfield B. D. (2010). Novel associations between pathogens, insects and tree species threaten world forests. N. Zeal. J. For. Sci. 40, S95–S104. Available online at: https://www.scionresearch.com/nzjfs

[B59] Yamaoka Y. Wingfield M. J. Takahashi I. Solheim H. (1997). Ophiostomatoid fungi associated with the spruce bark beetle Ips typographus f. aponicus in Japan. Mycol. Res. 101, 1215–1227. 10.1017/S0953756297003924

[B60] Yin M. Duong T. A. Wingfield M. J. Zhou X. De Beer Z. W. (2015). Taxonomy and phylogeny of the *Leptographium procerum* complex, including *Leptographium sinense*. sp. nov. and *Leptographium longiconidiophorum*. sp. nov. Antonie van Leeuwenhoek. 107, 547–563. 10.1007/s10482-014-0351-9 25510728

[B61] Zhou X. de Beer Z. W. Harrington T. C. McNew D. Kirisits T. Wingfield M. J. (2004). Epitypification of *Ophiostoma galeiforme* and phylogeny of species in the *O. galeiforme* complex. Mycologia 96, 1306–1315. 10.1080/15572536.2005.11832880 21148954

[B62] Zhou X. D. de Beer Z. W. Wingfield M. J. (2013). Ophiostomatoid fungi associated with conifer infecting bark beetles in China, in CBS-KNAW Fungal Biodiversity Centre, Ophiostomatoid Fungi: Expanding Frontiers, eds Seifert K. A. de Beer Z. W. Wingfield M. J. (Utrecht: CBS), 91–98.

[B63] Zhou X. D. Jacobs K. Morelet M. Ye H. Lieutier F. Wingfiled M. J. (2000). A new *Leptographium* species associated with *Tomicus piniperda* in South Western China. Mycoscience 41, 573–578 10.1007/BF02460923

