# Supplementary material for: Identification of ophiostomatalean fungi associated with Tomicus pilifer infesting Pinus koraiensis in Northeastern China
Source: Front Microbiol. 2022 Sep 2;13:919302. doi: 10.3389/fmicb.2022.919302 (PMC9479222; doi:10.3389/fmicb.2022.919302)
Supplement: Supplementary Table S1 — Diversity of ophiostomatalean fungi associated with Tomicus spp. [file Table_1.DOCX]

**Supplementary Table S1.** Diversity of ophiostomatalean fungi associated with *Tomicus* spp.

| Taxon | Species | Host | *Tomicus* spp. | Location | Reference |
| --- | --- | --- | --- | --- | --- |
| 1 | *Ceratocystiopsis minuta* | *Pinus sylvestris* | *Tomicus piniperda*, *T. minor* | Poland, Sweden | Mathiesen-Kaarik, 1953; Jankowiak, 2006; 2008 |
| 2 | *C. autographa* |  | *T. piniperda* | Czechoslovakia | Kotynkova-Sytchrova, 1966 |
| 3 | *Esteya vermicola* | *Pinus yunnanensis* | *T. yunnanensis* | China | Wang et al., 2019 |
| 4 | *Graphilbum anningense* | *P. yunnanensis* | *T. yunnanensis*, *T. minor* | China | Wang et al., 2019 |
| 5 | *Gra. fragrans* | *P. yunnanensis* | *T. minor* | China | Zhou et al., 2013 |
| 6 | *Gra. pseudormiticum* | *P. sylvestris* | *T. piniperda*, *T. minor* | Austria | Jankowiak, 2006; Jacobs et al., 2003 |
| 7 | *Gra. pycnocephalum* | *P. sylvestris* | *T. piniperda*, *T. minor* | Poland | Jankowiak, 2006; 2008 |
| 8 | *Grosmannia galeiformis* | *P. sylvestris* | *T. piniperda* | Chile, Africa, South, Sweden | Zhou et al., 2004 |
| 9 | *G. huntii* | *P. sylvestris*, *Pinus thunbergii* | *T. piniperda* | Britain | Gibbs and Inman, 1991 |
| 10 | *G. koreana* | *Pinus densiflora*, *Pinus koraiensis* | *T. piniperda* | Korea | Kim et al., 2005 |
| 11 | *G. piceiperda* | *P. sylvestris* | *T. piniperda* | England, France | Jankowiak, 2006; Solheim and Långström, 1991; Kirisits and Grubelnik, 2000 |
| 12 | *Leptographium euphyes* | *Pinus strobus, Pinus radiata* | *T. piniperda* | New Zealand | Jacobs and Wingfield., 2001 |
| 13 | *L. guttulatum* | *P. sylvestris* | *T. piniperda*, *T. minor*, *T. destruens* | Austria, England, France | Kirisits and Grubelnik, 2000; Jacobs and Wingfield, 2001; Jacobs et al., 2001; Sabbatini et al., 2006 |
| 14 | *L. japonicus* | Japanese red pines | *T. piniperda*, *T. minor* | Japan | Masuya et al., 1999a; b |
| 15 | *L. koreanum* | *Pinus densiflora* | *T. piniperda* | Korea | Kim et al. 2005 |
| 16 | *L. lundbergii* | *P. sylvestris*, *P. thunbergia*, *Pinus pinea*, Japanese red pines | *T. piniperda*, *T. minor*, *T. destruens* | Poland, Britain, Italy, Japan | Jankowiak, 2006; 2008; Gibbs and Inman, 1991; Jacobs and Wingfield, 2001; Sabbatini et al., 2006 |
| 17 | *L. panxianense* | *P. armandii*, *P. yunnanensis* | *T. armandii*, *T. yunnanensis* | China | Pan et al. 2020b |
| 18 | *L. pini*-*densiflorae* | *P. densiflora* | *T. piniperda* | Japan | Masuya et al., 1999b |
| 19 | *L. procerum* | *P. sylvestris*, *P. thunbergia*, Japanese red pines | *T. piniperda*, *T. minor* | Poland, Britain, Japan | Jankowiak, 2006; 2008; Gibbs and Inman, 1991; Masuya et al., 1999a; b |
| 20 | *L. puerense* | *P. kesiya* | *T. minor* | China | Pan et al., 2020b |
| 21 | *L. serpens* | *Pinus pinea* | *T. destruens* | Italy | Sabbatini et al., 2006 |
| 22 | *L. sinense* | *Pinus armandii* | *T. armandii* | China | Pan et al. 2018a; Yin et al., 2015 |
| 23 | *L. wingfieldii* | *P. sylvestri*, *Pinus thunbergia*, Japanese red pines, *Pinus pinea* | *T. piniperda*,  *T. destruens* | Poland, Italy, France, Britain, Sweden, Japan | Jankowiak, 2006; Gibbs and Inman, 1991; Sabbatini et al., 2006; Masuya et al., 1999a; b; Lieutier et al., 1989; Ben Jamaa et al., 2007; |
| 24 | *L. wushanense* | *Pinus armandii* | *T. armandii* | China | Pan et al., 2020a |
| 25 | *L. yunnanensis* | *P. yunnanensis*, *P. kesiya* | *T. yunnanensis*,  *T. brevipilosus* | China | Wang et al., 2019; Zhou et al., 2000 |
| 26 | *Leptographium* sp. | Japanese red pines | *T. piniperda*, *T. minor* | Japan | Masuya et al., 1999a; b |
| 27 | *Ophiostoma aggregatum* | *P. yunnanensis* | *T. yunnanensis*,  *T. minor* | China | Wang et al., 2019 |
| 28 | *O. brevipilosi* | *P. kesiya* | *T. brevipilosus* | China | Wang et al., 2019; Chang et al., 2017 |
| 29 | *O. brunneo*-*ciliatum* | *P. sylvestris* | *T. minor* | Russia | Linnakoski et al. 2010 |
| 30 | *O. canum* | *P. sylvestris*, Japanese red pines, *P. yunnanensis* | *T. piniperda*, *T. minor*, *T. yunnanensis* | Poland, Sweden, France, Japan, China | Mathiesen-Kaarik, 1953; Jankowiak, 2006; 2008; Wang et al., 2019; Solheim and Långström, 1991; |
| 31 | *O. canum*-like | *P. sylvestris* | *T. piniperda* | Russia | Linnakoski et al., 2010 |
| 32 | *O. clavatum* |  | *T. piniperda* | Sweden | Mathiesen-Kaarik, 1953; Masuya et al., 1999a; b |
| 33 | *O. floccosum* | *P. sylvestris* | *T. minor* | Sweden, Japan | Mathiesen-Kaarik, 1953; Masuya et al., 1999a; b |
| 34 | *O. ips* | Japanese red pines, *P. yunnanensis*, *Pinus pinea* | *T. piniperda*,  *T. yunnanensis*,  *T. destruens* | Sweden, Japan, Italy, China | Mathiesen-Kaarik, 1953; Zhou et al., 2013; Masuya et al., 1999a; b; Ben Jamaa et al., 2007 |
| 35 | *O. karelicum* | *P. sylvestris* | *T. piniperda*, *T. minor* | Russia, Finland | Linnakoski et al., 2010 |
| 36 | *O. minus* | *P. sylvestris*, Japanese red pines, *P. yunnanensis*, *Pinus pinea* | *T. piniperda*, *T. minor*, *T. yunnanensis*,  *T. destruens* | Poland, France, Sweden, Russia, Italy, Japan, China, Finland | Mathiesen-Kaarik, 1953; Jankowiak, 2006; 2008; Wang et al., 2019; Solheim and Långström, 1991; Masuya et al., 1999a; b; Lieutier et al., 1989; Ben Jamaa et al., 2007 |
| 37 | *O. minutum* |  | *T. piniperda*, *T. minor* | Sweden | Mathiesen-Kaarik, 1953; Masuya et al., 1999a; b |
| 38 | *O. piceae* | *P. sylvestris*, Japanese red pines | *T. piniperda*, *T. minor* | Poland, Sweden, Japan | Jankowiak, 2006; 2008; Masuya et al., 1999a; b; Linnakoski et al., 2010 |
| 39 | *O. piceaperdum* | *P. sylvestris* | *T. piniperda* | Poland | Jankowiak, 2006; |
| 40 | *O. piliferum* | *P. sylvestris*, Japanese red pines | *T. piniperda*, *T. minor* | Sweden, Poland, Japan | Mathiesen-Kaarik, 1953; Jankowiak, 2006; Masuya et al., 1999a; b; Linnakoski et al., 2010 |
| 41 | *O. pluriannulatum* |  | *T. minor* | Sweden | Mathiesen-Kaarik, 1953; Masuya et al., 1999b |
| 42 | *O. quercus* | *P. yunnanensis* | *T. yunnanensis* | China | Zhou et al., 2013 |
| 43 | *O. tingens* | *P. yunnanensis*, *P. sylvestris* | *T. piniperda*, *T. minor*  *T. yunnanensis*, | France, Sweden, China | Mathiesen-Kaarik, 1953; Wang et al., 2019; Mathiesen, 1950; Rennerfelt, 1950; Pan et al. 2017 |
| 44 | *Ophiostoma* sp. 1(1) | Japanese red pines | *T. piniperda*, *T. minor* | Japan | Masuya et al., 1999a; b |
| 45 | *Ophiostoma* sp. 1(2) | *P. yunnanensis* | *T. yunnanensis* | China | Wang et al., 2019 |
| 46 | *Ophiostoma* sp. 2 | Japanese red pines | *T. piniperda*, *T. minor* | Japan | Masuya et al., 1999a; b |
| 47 | *Ophiostoma* sp. I | *Picea abies* | *T. minor* | Finland | Linnakoski et al., 2010 |
| 48 | *Ophiostoma* sp. N | *P. sylvestris* | *T. minor* | Russia | Linnakoski et al., 2010 |
| 49 | *Sporothrix abietina* | *P. yunnanensis* | *T. yunnanensis* | China | Zhou et al., 2013 |
| 50 | *S. macroconidia* | *P. yunnanensis*, *P. kesiya* | *T. yunnanensis*,  *T. brevipilosus* | China | Wang et al., 2019 |
| 51 | *S. nebularis* | *P. yunnanensis* | *T. yunnanensis* | China | Chang et al. 2017 |
| 52 | *S. pseudoabietina* | *P. yunnanensis* | *T. yunnanensis*,  *T. minor* | China | Wang et al., 2019 |

**Reference**

Ben Jamaa, M.L.; Lieutier, F.; Yart, A.; Jerraya, A.; Khouja, M. L. (2007) The virulence of phytopathogenic fungi associated with the bark beetles *Tomicus piniperda* and *Orthotomicus erosus* in Tunisia. *For. Pathol*, 37, 51-63. https://doi.org/10.1111/j.1439-0329.2007.00478.x

Chang, R.; Duong, T.A.; Taerum, S.J.; Wingfield, M.J.; Zhou, X.; De Beer, Z.W. (2017) Ophiostomatoid fungi associated with conifer-infesting beetles and their phoretic mites in Yunnan, China. *MycoKeys*, (28), 19. doi:10.3897/mycokeys.28.21758

Gibbs, J.N.; Inman, A. (1991) The pine shoot beetle *Tomicus piniperda* as a vector of blue stain fungi to windblown pine, *Forestry*, 64 239-249.

Jacobs, K.; Wingfield, M.J. (2001) *Leptographium* Species: Tree Pathogens, Insect Associates, and Agents of Blue Stain. *American Phytopathological Society Press, St. Paul*, https://doi.org/10.1093/forestscience/48.4.791

Jacobs, K.; Wingfield, M.J.; Coetsee, C.; Kirisits, T.; Wingfield, B.D. (2001) *Leptographium guttulatum* sp. nov., a new species from spruce and pine in Europe. *Mycologia*, 93(2), 380-388. https://doi.org/10.1080/00275514.2001.12063169

Jacobs, K.; Kirisits, T.; Wingfield, M.J. (2003) Taxonomic re-evaluation of three related species of *Graphium*, based on morphology, ecology and phylogeny. *Mycologia*, 95, 714-727. https://doi.org/10.1080/15572536.2004.11833075

Jankowiak, R. (2008) Fungi associated with *Tomicus minor* on *Pinus sylvestris* in Poland and their succession into the sapwood of beetle-infested windblown trees. *Canadian journal of forest research*, 38(10): 2579-2588. https://doi.org/10.1139/X08-101

Jankowiak, R. (2006) Fungi associated with *Tomicus piniperda* in Poland and assessment of their virulence using Scots pine seedlings. *Annals of forest science*, 63(7): 801-808. DOI: 10.1051/forest:2006063

Kim, J.J.; Lim, Y.W.; Breuil, C.; Wingfield, M.J.; Zhou, X.D.; Kim, G.H. (2005) A new *Leptographium* species associated with *Tomicus piniperda* infesting pine logs in Korea. *Mycol Res*, 109: 275-284. DOI: https://doi.org/10.1017/S0953756204002060

Kirisits, T.; Grubelnik, R.; Fuhrer, E. (2000) Die okologische Bedeutung von Blauepilzen fur rindenbrutende Borkenkafer. The ecological role of blue-stain fungi for phloem-feeding bark beetles. In Mariabrunner Waldbautage 1999-Umbau sekundarer Nadelwalder. Edited by F. Muller. Schriftenreihe der Forstlichen Bundesversuchsanstalt Wien, Vienna. FBVA-Berichte, 111. pp. 117-137.

Kotynkova-Sytchrova, E. (1966) The mycoflora of bark beetle galleries in Czecholslovakia. *Ceska Mykologie*, 20, 45-53.

Lieutier, F.; Yart, A.; Garcia, J.; Ham, M.C.; Morelet, M.; Levieux, J. (1989) Champignons phytopathogènes associés à deux coléoptères scolytidae du pin sylvestre (*Pinus sylvestris* L.) et étude préliminaire de leur agressivité envers l’hôte, *Ann. Sci. For*, 46 201–216 (in French with English summary)

Linnakoski, R.; De Beer, Z.W.; Ahtiainen, J.; Sidorov, E.; Niemelä, P.; Pappinen, A.; Wingfield, M.J. (2010) *Ophiostoma* spp. associated with pine-and spruce-infesting bark beetles in Finland and Russia. *Persoonia: Molecular Phylogeny and Evolution of Fungi* 25, 72. DOI: https://doi.org/10.3767/003158510X550845

Masuya, H.; Kaneko, S.; Yamaura, Y.; Yamaoka, Y. (1999a) Ophiostomatoid fungi isolated from Japanese red pine and their relationships with bark beetles. *Mycoscience*, 50(3), 212-223.

Masuya, H.; Kaneko, S.; Yamaoka, Y.; Osawa, M. (1999b) Comparisons of ophiostomatoid fungi associated with *Tomicus piniperda* and *T. minor* in Japanese red pine. *Journal of Forest Research*, 4(2), 131-135.

Mathiesen, A. (1950) Über einige mit Borkenkäfern assoziierten Bläuepilze in Schweden. *Oikos*, 2, 275-308.

Mathiesen-Kaarik, A. (1953) Eine Ubersicht uber die gewohnlichsten mit Borkenkafern assoziierten Blauepilze in Schweden und einige fur Schweden neue Blauepilze. *Meddelanden fran Statens Skogforskningsinstitut*, 43, 1-74.

Pan, Y., Lu, J., Zhou, X. D., Chen, P., Zhang, H., and Ye, H. (2020a). *Leptographium wushanense* sp. nov., associated with *Tomicus armandii* on *Pinus armandii* in Southwestern China. Mycoscience 61(1), 43–48. doi: 10.1016/j.myc.2018.10.003

Pan, Y.; Lu, J.; Zhou, X.D.; Yu, Z. F.; Chen, P.; Wang, J.; Ye, H. (2020b) Two new species of *Leptographium* associated with *Tomicus* spp. infesting *Pinus* spp. in Southwestern China. International Journal of Systematic and Evolutionary *Microbiology*, 70(8), 4798-4807.

Pan, Y.; Lu, J.; Zhou, X.D.; Chen, P.; Zhang, H.; Ye, H. (2018a) *Leptographium wushanense* sp. nov., associated with Tomicus armandii on *Pinus armandii* in Southwestern China. *Mycoscience*, 61(1), 43-48. DOI: 10.1016/j.myc.2018.10.003

Pan, Y.; Zhao, T.; Krokene, P.; Yu, Z.F.; Qiao, M.; Lu, J.; Ye, H. (2018b) Bark Beetle-Associated Blue-Stain Fungi Increase Antioxidant Enzyme Activities and Monoterpene Concentrations in *Pinus yunnanensis*. *Frontiers in plant science*, 9:1731. https://doi.org/10.3389/fpls.2018.01731

Pan, Y.; Chen, P.; Lu, J.; Zhou, X.D.; Ye, H. (2017) First report of blue-stain in *Pinus yunnanensis* caused by *Ophiostoma tingens* associated with *Tomicus minor* in China. *Journal of Plant Pathology*. 99(3). DOI: http://dx.doi.org/10.4454/jpp.v99i3.3931

Rennerfelt, E. (1950) Über den Zusammenhang zwischen dem Verblauen des Holzes und den Insekten. *Oikos*, 120-137.

Sabbatini, P. G.; Capretti, P.; Tiberi, R. (2006) Associations between *Tomicus destruens* and *Leptographium* spp. in *Pinus pinea* and *P. pinaster* stands in Tuscany, central Italy. *Forest Pathology*. 36, 14-20. https://doi.org/10.1111/j.1439-0329.2006.00427.x

Solheim, H.; Långström, B. (1991) Blue-stain fungi associated with *Tomicus piniperda* in Sweden and preliminary observations on their pathogenicity. *Annales des sciences forestières*. 48: 149-156. DOI: https://doi.org/10.1051/forest:19910203

Wang, H.M.; Wang, Z.; Liu, F.; Wu, C.X.; Zhang, S.F., Kong, X.B. et al. (2019) Differential patterns of ophiostomatoid fungal communities associated with three sympatric *Tomicus* species infesting pines in south-western China, with a description of four new species. *MycoKeys*, 50, 93. doi: 10.3897/mycokeys.50.32653. eCollection 2019

Yin, M.; Duong, T.A.; Wingfield, M.J.; Zhou, X.; De Beer, Z.W. (2015) Taxonomy and phylogeny of the *Leptographium procerum* complex, including *Leptographium sinense*. sp. nov. and *Leptographium longiconidiophorum*. sp. nov. *Antonie van Leeuwenhoek*. 107(2):547-563

Zhou, X.D.; de Beer, Z.W.; Wingfield, M.J. (2013) Ophiostomatoid fungi associated with conifer infecting bark beetles in China. In: Seifert, K.A.; de Beer, Z.W.; Wingfield, M.J. (Eds) Ophiostomatoid fungi: Expanding frontiers. CBS, Utrecht, The Netherlands. 91-98.

Zhou, X.; de Beer, Z.W.; Harrington, T. C.; McNew, D.; Kirisits, T.; Wingfield, M.J. (2004) Epitypification of *Ophiostoma galeiforme* and phylogeny of species in the *O.* *galeiforme* complex. *Mycologia*. 96(6), 1306-1315. https://doi.org/10.1080/15572536.2005.11832880

Zhou, X.D.; Jacobs, K.; Morelet, M.; Ye, H.; Lieutier, F.; Wingfiled, M.J. (2000) A new *Leptographium* species associated with *Tomicus piniperda* in South Western China. *Mycoscience*. 41: 573-578 https://doi.org/10.1007/BF02460923
