# Supplementary material for: Identification of ophiostomatalean fungi associated with Tomicus pilifer infesting Pinus koraiensis in Northeastern China
Source: Front Microbiol. 2022 Sep 2;13:919302. doi: 10.3389/fmicb.2022.919302 (PMC9479222; doi:10.3389/fmicb.2022.919302)
Supplement: Supplementary Table S2 — Reference sequences which are involved in phylogenetic tree in this study. [file Table_2.DOCX]

**Table S2.** Reference sequences which are involved in phylogenetic tree in this study.

| **Species** | **Isolate number** | **Type** | **Isolated from** | **Country** | **Collector** | **GenBank Accession Numbers** | | |
| --- | --- | --- | --- | --- | --- | --- | --- | --- |
|  |  |  |  |  |  | BT | EF | ITS/ITS2-LSU |
| *Cop. brevicomis* | CMW40952 | T | *Dendroctonus brevicomis* | USA | T. Harrington | EU913761 | - | EU913722 |
| *Cop. brevicomis* | CBS 333.97(UM 1452) |  | *Dendroctonus brevicomis* | USA | T. Harrington | EU913761 | - | EU913722 |
| *Cop. collifera* | CBS 126.89 |  | - | - | D. Vu | - | - | MH862160 |
| *Cop. collifera* | CMW7074 |  | *Dendroctonus valens* on *Pinus teocote* | Mexico | J. Marmolejo | EU913760 | - | EU913721 |
| *Cop. longispora* | UM 48 |  | *Pinus* sp. | Canada | A. Olchowecki | EU913762 | - | EU913723 |
| *Cop. lunata* | CMW55898 |  | *Xylosandrus crassiusculus* | South Africa | W.J. Nel | MW066755 | - | MW028170 |
| *Cop. lunata* | CMW55897 | T | *Xylosandrus crassiusculus* | South Africa | W.J. Nel | MW066754 | - | MW028169 |
| *Cop. manitobensis* | UM214 |  | Unknown | Canada | J. Reid | EU913754 | - | EU913715 |
| *Cop. manitobensis* | UM237 |  | Galleries of *Dendroctonus ponderosae* | Canada | J. Reid | EU913753 | - | EU913714 |
| *Cop. minima* | UM1501 |  | Galleries of *Dendroctonus ponderosae* | Canada | J. Reid | EU913742 | - | EU913703 |
| *Cop. minima* | UM235 |  | Galleries of *Dendroctonus ponderosae* | Canada | J. Reid | EU913741 | - | EU913702 |
| *Cop. minima* | UM85 |  | Galleries of *Dendroctonus ponderosae* | Canada | J. Reid | EU913740 | - | EU913701 |
| *Cop. minuta* | RJ5095 (UM1533) |  | *Ips typographus* | Poland | R. Jankowiak | EU913737 | - | EU913698 |
| *Cop. minuta* | CBS 116796 |  | Sapwood of *Ips typographus* | Poland | T. Kirisits | EU913734 | - | EU913695 |
| *Cop. minuta* | RJ705 (UM1532) |  | *Ips typographus* | Poland | R. Jankowiak | EU913736 | - | EU913697 |
| *Cop. minuta* | YCC139 |  | Adult beetle of Ips typographus japonicus | Japan | Y. Yamaoka | EU913732 | - | EU913693 |
| *Cop. minuta* sp. 2 | CBS116795 |  | Perithecia in galleries | Poland | T. Kirisits | EU913727 | - | EU913688 |
| *Cop. minuta* sp. 3 | CBS463.77(UM846) |  | Log | USA | I. Ritos | EU913725 | - | EU913686 |
| *Cop. minuta-bicolor* | CBS635.66(UM844) |  | Galleries of *Ips* sp. | USA | R.W. Davidson | EU913745 | - | EU913706 |
| *Cop. minuta-bicolor* | UM480 |  | Galleries of bark beetles | Canada | J. Reid | EU913744 | - | EU913705 |
| *Cop. neglecta* | CBS 100596 | T | - | - | D.Vu | - | - | MH862711 |
| *Cop. pallidobrunnea* | WIN(M) 51 |  | *Populus tremuloides* | Duck Mountain | B. Strzałka | MN901013 | MN901028 | MN901004 |
| *Cop. ranaculosa* | CBS216.88 | T | Tree infested with *Dendroctonus frontalis* | USA | J.R. Bridges | EU913752 | - | EU913713 |
| *Cop. rollhanseniana* | UM113 |  | Beetle galleries of standing tree | Norway | J. Reid | EU913757 | - | EU913718 |
| *Cop. rollhanseniana* | UM110 |  | Beetle galleries of standing tree | Norway | J. Reid | EU913758 | - | EU913719 |
| *Cop. synnemata* | KFL16216DA |  | *Dryocoetes alni* | Poland | B. Strzałka | MN901005 | MN901014 | MN900984 |
| *Cop. synnemata* | KFL17718DA |  | *Dryocoetes alni* | Paprocice | B. Strzałka | MN901010 | MN901019 | MN900989 |
| *Cop. weihaiensis* | SNM649 | T | Gallery | China | R. Chang | MZ019525 | MZ853082 | MW989413 |
| *Cop. weihaiensis* | SNM634 |  | Gallery | China | R. Chang | MZ019524 | MZ853081 | MW989412 |
| *Cop. weihaiensis* | SNM649 | T | Gallery | China | R. Chang | MZ019525 | MZ853082 | MW989413 |
| *Cop. yantaiensis* | SNM582 |  | Gallery | China | R. Chang | MZ019522 | MZ853079 | MW989410 |
| *Cop. yantaiensis* | SNM650 | T | Gallery | China | R. Chang | MZ019523 | MZ853080 | MW989411 |
| *Ceratocystiopsis sp.* | VPRI43834 |  | *Ipis grandicollis* | New South Wales | A. J. Carnegie, C.Trollip | MW066350 | MW066396 | MW046062 |
| *Ceratocystiopsis sp.* | CBS145.59 |  | Unknown | USA | R.W. Davidson | EU913726 | - | EU913687 |
| *Ceratocystiopsis sp.* | VPRI43835 |  | *Ipis-grandicollis* | New South Wales | A. J. Carnegie, C.Trollip | MW066351 | MW066397 | MW046063 |
| *Ceratocystiopsis sp.* | VPRI43836 |  | *Ipis-grandicollis* | New South Wales | A. J.Carnegie, C.Trollip | MW066352 | MW066398 | MW046064 |
| *Ceratocystiopsis* sp. 1 | Cop. sp. 1ii |  | Log infested with *Dendroctonus ponderosae* | British Columbia,Canada | J.-J. Kim | EU913747 | - | EU913708 |
| *Ceratocystiopsis* sp. 1 | Cop. sp. 1iii |  | Log infested with *Dendroctonus ponderosae* | British Columbia,Canada | J.-J. Kim | EU913748 | - | EU913709 |
| *Ceratocystiopsis* sp. 2 | YCC329 |  | Adult beetle of *Ips subelongatus* | Japan | Y. Yamaoka | EU913750 | - | EU913711 |
| *Ceratocystiopsis* sp. 2 | YCC330 |  | Adult beetle of *Ips subelongatus* | Japan | Y. Yamaoka | EU913749 | - | EU913710 |
| *Ceratocystiopsis* sp. 2 | YCC294(JCM 9368) |  | Gallery of *Ips subelongatus* | Japan | Y. Yamaoka | EU913730 | - | EU913691 |
| *Ceratocystiopsis* sp. 3 | Cop. sp3ii (SWT3) |  | Body of *Ips perturbatus* | Canada | S. M. Alamouti | EU913756 | - | EU913717 |
| *Grosmannia* *penicillata* | CMW2642 |  | *Ips typographus* | Sweden | - | MN647820 | MN647909 | MN644478 |
| *G. piceiperda* | C274 |  | Unknown | USA | T.C. Harrington | AY707195 | - | AY707209 |
| *Graphilbum acuminatum* | CMW54769 | T | *Ips acuminatus* | Mircze | - | MN548938 | MN548952 | MN548902 |
| *Gra. anningense* | CFCC52631 | T | - | China | H.M. Wang | MH683595 | - | MH555903 |
| *Gra. anningense* | CFCC52632 |  | - | China | H.M. Wang | MH683596 | - | MH555901 |
| *Gra. carpaticum* | 143aMFJD | T | *Abies alba* | Poland | R. Jankowiak | - | - | KY568116 |
| *Gra. acuminatum* | CMW54769 | T | *Ips acuminatus* | Mircze | R. Jankowiak | MN548938 | MN548952 | MN548902 |
| *Gra. acuminatum* | CMW44771 |  | *Pityogenes bidentatus* | Bendiksmyra | R. Jankowiak | MN548933 | MN548947 | MN548897 |
| *Gra. carpaticum* | CMW43141 |  | *Pinus piceae* | Nawojowa | R. Jankowiak | KY568224 | MN548956 | KY568116 |
| *Gra. carpaticum* | CMW43142 | T | *Cryphalus piceae* | Nawojowa | R. Jankowiak | KY568226 | MN548953 | KY568114 |
| *Gra. crescericum* | CBS130864 | T | *Hylurgops palliatus* | Spain | - | Not obtained | MN548978 | MN548925 |
| *Gra. crescericum* | CMW54785 |  | *Hylurgops palliatus* | Bardufoss | R. Jankowiak | Not obtained | MN548984 | MN548931 |
| *Gra. crescericum* | CBS130866 |  | *Orthotomicus erosus* | Spain | R. Jankowiak | Not obtained | MN548979 | MN548926 |
| *Gra. curvidentis* | 55KFJD |  | *Abies alba* | Poland | P. Bilanski | - | KY568502 |  |
| *Gra. curvidentis* | 49aKFJD |  | *Abies alba* | Poland | P. Bilanski | - | KY568501 |  |
| *Gra. curvidentis* | 55KFJD | T | *Abies alba* | Poland | P. Bilanski | - | - | KY568111 |
| *Gra. fragrans* | C1224 |  | - | Sweden | T.C. Harrington | - | - | AF198248 |
| *Gra. furuicola* | CMW44770 |  | *Tomicus piniperda* | Stengelsen | - | Not obtained | MN548961 | MN548907 |
| *Gra. furuicola* | CMW54766 | T | *Ips sexdentatus* | Leium | R. Jankowiak | Not obtained | MN548960 | MN548906 |
| *Gra. gorcense* | CMW34153 |  | *Tetropium* sp. | Poland | R. Jankowiak | MN548945 | MN548972 | MN548919 |
| *Gra. gorcense* | CMW34151 |  | *Tetropium* sp. | Poland | R. Jankowiak | MN548943 | MN548970 | MN548917 |
| *Gra. gorcense* | CMW34153 | T | *Tetropium* sp. | Poland | R. Jankowiak | MN548945 | MN548972 | MN548919 |
| *Gra. interstitiale* | CMW54780 |  | *Hylurgops interstitialis* | Russia | R. Jankowiak | Not obtained | MN548963 | MN548909 |
| *Gra. interstitiale* | CBS145817 |  | *Hylurgops interstitialis* | Russia | R. Jankowiak | Not obtained | MN548964 | MN548910 |
| *Gra. interstitiale* | CMW54782 |  | *Hylurgops interstitialis* | Russia | R. Jankowiak | Not obtained | MN548966 | MN548912 |
| *Gra. ipis-grandicollis* | VPRI43762 | T | *Pinus radiata* | Moss Vale, NSW | A.J. Carnegie | MW066359 | MW066405 | MW046071 |
| *Gra. ipis-grandicollis* | VPRI43760 |  | *Pinus radiata* | Tumut, NSW | D. Sargeant | MW066357 | MW066403 | MW046069 |
| *Gra. ipis-grandicollis* | VPRI43761 | M | *Pinus radiata* | Moss Vale, NSW | A.J. Carnegie | MW066358 | MW066404 | MW046070 |
| *Gra. kesiyae* | CMW41729 | T | *P. kesiya/ P. szemaoensis* | China | R. Chang | MG205713 | - | MG205669 |
| *Gra. kesiyae* | CMW41703 |  | *P. kesiya/ P. szemaoensis* | China | R. Chang | MG205712 | - |  |
| *Gra. kesiyae* | CMW41686 |  | *P. kesiya/ P. szemaoensis* | China | R. Chang | MG205711 | - | =MG205668 |
| *Gra. kesiyae* | CMW41774 |  | *P. kesiya/ P. szemaoensis* | China | R. Chang | MG205714 | - | MG205668 |
| *Gra. microcarpum* | CMW11778 |  | - | China | R. Chang | MG205710 | - |  |
| *Gra. microcarpum* | 94aLMD |  | - | Western Carpathians | R. Jankowiak | KY568222 | - |  |
| *Gra. microcarpum* | YCC612 |  | *Ips subelongatus* | Japan | T.-C.Lin | - | - | GU134170 |
| *Gra. nigrum* | CBS 163.61 |  | - | USA | R.W. Davidson | - | - | MH858010 |
| *Gra. parakesiyea* | CFCC54515, CXY2540 |  | *Dendroctonus armandi* | China | H.M. Wang | MW770446 | - | MW459987 |
| *Gra. parakesiyea* | CFCC54514, CXY2539 |  | *Dendroctonus armandi* | China | H.M. Wang | MW770445 | - | MW459986 |
| *Gra. parakesiyea* | CFCC53924, CXY2516 | T | *Dendroctonus armandi* | China | H.M. Wang | MW770444 | - | MW459985 |
| *Gra. puerense* | CMW41942 | T | *P. kesiya /I. acuminatus* | China | R. Chang | MG205719 | - | MG205671 |
| *Gra. puerense* | CMW41671 |  | *P. kesiya /I. acuminatus* | China | R. Chang | MG205717 | - | =MG205671 |
| *Gra. puerense* | CMW41667 |  | *P. kesiya /I. acuminatus* | China | R. Chang | MG205716 | - | MG205670 |
| *Gra. puerense* | CMW41619 |  | *P. kesiya /I. acuminatus* | China | R. Chang | MG205715 | - | MG205670 |
| *Gra. puerense* | CMW41942 | T | *P. kesiya /I. acuminatus* | China | R. Chang | MG205719 | - | MG205671 |
| *Gra. rectangulosporium* | C2477 |  | *Hylurgus ligniperda* | USA | S. Kim | - | - | GU129987 |
| *Gra. sexdentatum* | CMW54773/CBS145814^T^/N2015-1084/2/5/2 | T | *Ips sexdentatus* | Leium | R. Jankowiak | Not obtained | MN548968 | MN548915 |
| *Gra. sexdentatum* | CBS145815/N2015-1088/2/2 |  | *Ips sexdentatus* | Leium | R. Jankowiak | Not obtained | MN548967 | MN548913 |
| *Gra. sparsum* | CMW50158 | T | Bark beetle gallery | Fairbanks | R. Jankowiak | MN548946 | MN548977 | MN548924 |
| *Gra. sparsum* | CMW54772 |  | *P. subopacus* | Tølløvtjønna | R. Jankowiak | Not obtained | MN548976 | MN548923 |
| *Gra. tsugae* | UAMH 11701 |  | *Tsuga heterophylla* | Canada | James Reid | - | - | KJ661745 |
| *Gra.*cf. *rectangulosporium* | VPRI43843 |  | *Ipis-grandicollis* | Urbenville, New South Wales | A.J. Carnegie, C.Trollip | MW066361 | MW066407 | MW046073 |
| *Gra.*cf. *rectangulosporium* | VPRI43763 |  | *Ipis-grandicollis* | Rosewood, New South Wales | A.J. Carnegie | MW066360 | MW066406 | MW046072 |
| *Gra. fragrans* | VPRI43758 |  | *Ipis-grandicollis* | Rockley, New South Wales | D. Sargeant, | MW066353 | MW066401 | MW046067 |
| *Gra. fragrans* | CMW43200 |  | *Trypodendron lineatum* | Rozpucie | R. Jankowiak | KY568220 | KY568497 | KY568106 |
| *Gra.parakesiyea* | CFCC53924, CXY2516 | T | *Dendroctunus armandi* | China | H.M. Wang | MW770444 | - | MW459985 |
| *Gra.* cf. *rectangulosporium* | VPRI43761 | M | - | Moss Vale, NSW | A.J. Carnegie | MW066358 | MW066404 | MW046070 |
| *Graphilbum* sp. 1 | C2316 |  | *Hylurgus ligniperda* | USA | S. Kim | - | - | GU129997 |
| *Hawksworthiomyces lignivorus* | CMW18600 |  | Eucalyptus utility poles | Stellenbosch, SA | E. Meyer | EF139104 | - | EF127890 |
| *H. lignivorus* | CMW18598 |  | Eucalyptus utility poles | St Lucia, SA | E. Meyer | EF139102 | - | EF127888 |
| *Leptographium. abietinum* | CMW275 |  | *Picea engelmanni*i | Canada | K. Jacobs | - | OM631763 | OM501383 |
| *L. absconditum* | CMW39763 | T | *Orthotomicus laricis* on *Pinus nigra* | Spain | De Beer | - | OM631790 | OM501415 |
| *L. aenigmatica* | CMW2199 | T | *Tomicus piniperda*/bark beetles | North America | K. Jacobs | - | - | AY553389 |
| *L. alacris* | CMW 2844 | T | - | Portugal | T.A. Duong | - | - | JN135313 |
| *L. altius* | CMW12471 |  | *P. koraiensis* | China | D. Paciura | HQ406899 | HQ406875 | HQ406851 |
| *L. aureum* | ATCC16936 | T | Ascocarps in bark beetle-infested tree | Canada | R.C.R.-Jeffrey/R.W. | AY263187 | AY544633 | AY544610 |
| *L. celere* | CMW12422 | T | *Pinus kesiya* | China | D. Paciura | HQ406882 | HQ406858 | HQ406834 |
| *L. celere* | CMW12421 |  | *Pinus kesiya* | China | D. Paciura | HQ406883 | HQ406859 | HQ406835 |
| *L. clavigerum* | UAMH11148 |  | *Pinus contorta* wood from *Dendroctonus ponderosae* gallery | Canada | A.D. Roe | GU370215 | GU370258 | - |
| *L. clavigerum* | UAMH 11141 | T | *Dendroctonus ponderosae* larvae from *Pinus contorta* / *P. banksiana* hybrid | Canada | A.D. Roe | - | GU370247 | - |
| *L. clavigerum* | ATCC 18086 | T | Tree attacked by *Dendroctonus* sp. | Cache Creek, BC, Canada | R.C.R.-Jeffrey/R.W.Davidson | AY263194 | AY544636 | AY544613 |
| *L. conjunctum* | CMW41761 |  | *Pinus kesiya* | China | R. Chang | - | - | MG205679 |
| *L. conjunctum* | CMW12449 |  | *Pinus kesiya* | China | D. Paciura | HQ406880 | HQ406856 | HQ406832 |
| *L. conjunctum* | CMW12452 |  | *Pinus yunnanensis* / Hylurgops major | China | D. Paciura | HQ406881 | HQ406857 | HQ406833 |
| *L. conjunctum* | CMW12473 | T | *Pinus yunnanensis* / Hylurgops major | China | D. Paciura | HQ406879 | HQ406855 | HQ406831 |
| *L. cucullatum* | CMW44485 |  | *Ips typographus* | China | D. Paciura | MH124312 | MH124392 | MH144116 |
| *L. cucullatum* | CBS 218.83 | T | - | China | M. Villarreal | - | - | AJ538335 |
| *L. curviconidia* | CMW12425 | T | *P. koraiensis* / *Ips typographus* | China | D. Paciura | HQ406898 | HQ406874 | HQ406850 |
| *L. douglasii* | CMW2078 | T | *Tomicus piniperda*/bark beetles | North America | K. Jacobs | - | - | AY553381 |
| *L. galeiforme* | CMW5290 | T | logs of *Pinus radiata* | GBR | J.-J. Kim | - | - | AY744552 |
| *L. gracile* | CMW12398 | T | *Pinus armandii* / *Pissodes* sp. | China | D. Paciura | HQ406888 | HQ406864 | HQ406840 |
| *L. koreanum* | MCC206 | T |  | South Korea | - | - | - | OM501431 |
| *L. koreanum* | MUCL46335 |  | Sapwood underneath *D. valens* gallery in *P. tabuliformis* | China | Q. Lu | EU502810 | EU502825 | EU502798 |
| *L. koreanum* | KUC2102=MUCL 47422 |  | *P. koraiensis* log infesting by *T. piniperda* | Korea | J.-J. Kim | AY707184 | EU502827 | AY707197 |
| *L. koreanum* | MCC206 | T | - | Japan | - | - | - | AB222065 |
| *L. longiclavatum* | CBS120207 CAN |  | - | Canada | - | - | - | AY816686 |
| *L. lundbergii* | CMW2190 |  | *P. sylvestris* infested by *Pissodes pini* | Norway | F. Roll-Hansen | DQ062000 | DQ062033 | DQ062066 |
| *L. lundbergii* | CMW17264 | T | *P. sylvestris* | Sweden | A. Käärik | DQ062002 | DQ062035 | DQ062068 |
| *L. manifestum* | CMW12436 | T | *L. olgensis* / *I. subelongatus* | China | D. Paciura | HQ406887 | HQ406863 | HQ406839 |
| *L. manifestum* | CMW12461 |  | *P. yunnanensis* / *Polygraphus verrucifrons* | China | D. Paciura | HQ406886 | HQ406862 | HQ406838 |
| *L. neomexicanum* | CMW2079 |  |  | USA |  | AY534930 | AY536176 | AY553382 |
| *L. olivaceapini* | MUCL 18368 | T |  | USA | M. Villarreal | - | - | AJ538336 |
| *L. olivaceum* | CBS 138.51 | T |  | Finland | M. Villarreal | - | - | AJ538337 |
| *L. panxianense* | CGMCC3.20129 |  | *P. armandii* /*T. armandii* | China | Y. Pan | MH195238 | MH195244 | MH195232 |
| *L. panxianense* | CGMCC3.19690 | T | *P. armandii* /*T. armandii* | China | Y. Pan | MH195240 | MH195246 | MH195234 |
| *L. panxianense* | CGMCC 3.20127  3.20127 |  | *P. yunnanensis / T. yunnannesis* | China | Y. Pan | MG702076 | MG702103 | MG702049 |
| *L. pinicola* | CMW2398 | T | *P. resinosa* | Canada | J. Juzwik | DQ061994 | DQ062027 | DQ062060 |
| *L. pinicola* | CMW1874 |  | *P. densiflora* | Japan | M. J. Wingfield | DQ061996 | DQ062029 | DQ062062 |
| *L. procerum* | CMW 34542 | T | *D. valens* | USA | M. Yin | KM491374 | KM491483 | KM491423 |
| *L. puerense* | CGMCC3.19511 | T | *P. kesiya / T. minor* | China | Y. Pan | MH195241 | MH195247 | MH195235 |
| *L. puerense* | CGMCC3.20130 |  | *P. kesiya / T. minor* | China | Y. Pan | MG702078 | MG702105 | MG702051 |
| *L. pyrinum* | CMW169 | T | - | USA | K. Jacobs and others | DQ062006 | DQ062039 | DQ062072 |
| *L. qinlingense* | CFCC53940 |  | *D. armandi* | China | H. Wang | MW723027 | MW677129 | MW463381 |
| *L. qinlingense* | CFCC53941 | T | *D. armandi* | China | H. Wang | MW723028 | MW677130 | MW463382 |
| *L. radiaticola* | KUC2036 | T |  | China | X.D. Zhou | - | - | AY744551 |
| *L. reconditum* | CMW 15 | T | Zea mays rhizosphere | South Africa | W. Jooste | - | - | AF343690 |
| *L. shansheni* | CMW44462 | T | *Pinus koraiensis* | Heilongjiang, China | R. Chang | MH124293 | MH124373 | MH144097 |
| *L. serpens* | CMW 304 | T | - | Italy | G. Goidànich | JN135334 | JN135307 | JN135314 |
| *L. sinense* | CMW38172 | T | - | China | M. Yin, R. Chang,  X.D. Zhou | - | OM631825 | KM491419 |
| *L. sinoprocerum* | CMW29990 | T | *D. valens* | China | M. Yin | KM491383 | KM491492 | EU296773 |
| *L. sosnaicola* | CMW52084 | T | *Pinus sylvestris* | western Poland | R. Jankowiak | MT210359 | MT210397 | MT210337 |
| *L. sosnaicola* | CMW52083 |  | *Pinus sylvestris* | western Poland | R. Jankowiak | MT210361 | MT210399 | MT210339 |
| *L. sosnaicola* | CMW52081 |  | *Pinus sylvestris* | western Poland | R. Jankowiak | MT210360 | MT210398 | MT210338 |
| *L. terebrantis* | UAMH 11004 |  | *Dendroctonus ponderosae* larvae from *Pinus contorta* /*P. banksiana* hybrid | Canada | A. Rice | - | - | GU370281 |
| *L. truncatum* | CMW2850 |  | P. strobus | New Zealand | M. Dick | DQ061991 | DQ062024 | DQ062057 |
| *L. truncatum* | CMW644 |  | *P. sylvestris* infested by *Hylastes* | UK | J. Gibbs | DQ061992 | DQ062025 | DQ062058 |
| *L. truncatum* | CMW28 | T | *P. taeda* | South Africa | M. J. Wingfield | DQ061986 | DQ062019 | DQ062052 |
| *L. wageneri* | CMW2812 |  | *Pinus* sp. | USA | T.C. Harrington | - | - | AF343708 |
| *L. wingfieldii* | CMW2096 | T | - | FRA | - | - | - | AY553398 |
| *L. wushanense* | CFCC54525 |  | *D. armandi* | China | H.M. Wang | MW690922 | - | MW463386 |
| *L. wushanense* | CFCC54521 |  | *D. armandi* | China | H.M. Wang | MW723029 | MW677131 | MW463383 |
| *L. wushanense* | CFCC54522 |  | *D. armandi* | China | H.M. Wang | MW741823 | - | MW459990 |
| *L. wushanense* | CFCC53921 | T | *D. armandi* | China | H.M. Wang | MW741822 | - | MW459989 |
| *L. wushanense* | YMF1.04934 | T |  |  | - | - | MG878409 |  |
| *L. wushanense* | YMF1.04936 | T | *Pinus armandii* / *Tomicus armandii* | China, | Y. Pan | MG878408 | MG878409 | MG878407 |
| *L. yamaokae* | CMW4726 | T | - | JPN | - | - | - | JN135315 |
| *L. yunnanense* | CFCC52624 |  | *P. yunnanensis* / *Tomicus* sp. | China | H.M. Wang | MH603937 | MH606005 | MH487722 |
| *L. yunnanense* | CMW5304 |  | *P. yunnanensis* / *Tomicus* sp. | China | Y. Pan | AY534963 | AY536209 | AY553415 |
| *L. yunnanense* | CFCC52621 |  | *P. yunnanensis* / *Tomicus* sp. | China | H.M. Wang | MH603935 | MH606003 | MH487726 |
| *L. yunnanense* | CFCC52620 |  | *P. yunnanensis* / *Tomicus* sp. | China | H.M. Wang | MH603934 | MH606001 | MH487724 |
| *L. wushanense* | CFCC4524 |  | *P. yunnanensis* / *Tomicus* sp. | China | Y. Oan | - | - | MH487726 |
| *Masuyamyces acarorum* | CMW41850 | T | - | China | R. Chang | - | - | MG205657 |
| *M. massonianae* | CXY1610 | T | Trunk | China | H.M. Wang | - | - | KY094067 |
| *M. pallidulus* | CMW23278 | T | *Hylastes brunneus* | Finland | de Beer | HM031566 | - | HM031510 |
| *M. saponiodorus* | CMW29497 | T | *Ips typographus* | Finland | de Beer | HM031571 | - | HM031507 |
| *Ophiostoma adjuncti* | CMW135 | T | *Dendroctonus ponderosae* | USA | RW Davidson | - | - | AY546696 |
| *O. aggregatum* | CXY1876 | T | *Tomicus* species infesting pines | China | H.M. Wang | - | - | MH555894 |
| *O. ainoae* | CMW23123 |  | *Ips typographus* | Russia | Ahtiainen | HM031550 | KU094747 | HM031496 |
| *O. ainoae* | CMW1903 |  | - | Norway | Olsen | HM031553 | MG2057 | HM031495 |
| *O. ainoae* | CMW1037 | T | *Ips typographus* | Norway | H. Solheim | HM031552 | KU094745 | KU094682 |
| *O. angusticollis* | CBS186 |  | Pinus pinaster infested by *Tomicus piniperda* | Spain | M. Villarreal | - | - | AY924383 |
| *O. australiae* | CMW6606 | T | *Acacia mearnsii* | Australia | M. J. Wingfield | EF408606 | - | EF408603 |
| *O. bacillisporum* | MUCL 45378 |  | - | Belgium | F.-X. Carlier & T. Defrance | - | - | AY573258 |
| *O. bicolor* | CMW44472 |  | *P. koraiensis* | China | R. Chang | MH124284 | MH124367 | MH144088 |
| *O. bicolor* | CMW44598 |  | *P. koraiensis* | China | R. Chang | MH124286 | - | MH144090 |
| *O. bicolor* | CMW44599 |  | *P. koraiensis* | China | R. Chang | MH124287 | MH124368 | MH144091 |
| *O. bicolor* | CMW44471 |  | *P. koraiensis* | China | R. Chang | MH124283 | MH124366 | MH144087 |
| *O. bicolor* | CBS492.77 | T | Gallery of *Ips* sp. in *Picea glauca* | Colorado | S.M. Alamouti | DQ268635 | - | DQ268604 |
| *O. borealis* | CMW18966 | T | *Betula pubescens* | Norway | [G.K. Nkuekam](https://ifbicd85ae6022a1f4d78s6p6xucwn6quc6v6xfiac.eds.tju.edu.cn/wos/alldb/general-summary?queryJson=%5B%7B%22rowBoolean%22:null,%22rowField%22:%22AU%22,%22rowText%22:%22Nkuekam,%20Gilbert%20Kamgan%22%7D%5D&eventMode=oneClickSearch) | - | - | EF408593 |
| *O. brevipilosi* | CFCC52596 |  | - | China | - | MH619527 | - | - |
| *O. brevipilosi* | CMW41662 | T | - | China | R. Chang | MG205690 | MG2057 | MG205660 |
| *O. brevipilosi* | CFCC52597 |  | - | - | - | MH619528 | - | - |
| *O. breviusculum* | YCC 522 | T | *D. baikalicus* | Japan | Yamaoka | HM031517 | - | AB200423 |
| *O. brunneociliatum* | CMW 39842 |  | *I. sexdentatus* | Poland | R. Jankowiak | KU094691 | KU094751 | - |
| *O. brunneociliatum* | CMW 39829 |  | *I. cembrae* | Poland | R. Jankowiak | KU094690 | KU094752 | - |
| *O. brunneociliatum* | CMW5214 |  | - | Scotland | Kirisits | HM031558 | - | HM031501 |
| *O. brunneolus* | CMW23143 |  | *I. typographus* | Russia | J. Ahtiainen | HM031554 | KU094755 | KU094684 |
| *O. canum* | CBS133.51 | T | *Pinus sylvestris* | Sweden | Mathiesen | HM031518 | - | HM031489 |
| *O. catonianum* | C1084 | T | *Pyrus* sp. | Italy | Goidanich | - | - | AF198243 |
| *O. clavatum* | CMW41043 |  | *I. acuminatus* | Buskerud, Norway | H. Solheim | KU094712 | KU094763 | KU094712 |
| *O. clavatum* | CMW37983 | T | *Ips acuminatus* | Sweden | C. Villari | KU094705 | KU094759 | KU094685 |
| *O. coronatum* | CBS497.77 |  | - | Canada | M. Villarreal | - | - | AY924385 |
| *O. denticiliatum* | CMW29493 | T | *Scolytus ratzeburg*i infesting birch | Norway | R. Linnakoski | FJ804502 | - | FJ804490 |
| *O. distortum* | DSMZ4897 | T | *Abies concolor* | USA | M. Villarreal | - | - | AY924386 |
| *O. flexuosum* | CBS208.83 | T | *Picea abies* | Norway | M. Villarreal | - | - | AY924387 |
| *O. floccosum* | C1086 | T | - | Sweden | Kåårik | - | - | AF198231 |
| *O. fuscum* | CMW28019 |  | *Pityogenes chalcographus* | Russia | Linnakoski | HM031564 | - | HM031503 |
| *O. fuscum* | CMW23195 |  | *I. typographus* | Russia | Linnakoski | HM031565 | - | - |
| *O. fuscum* | CMW23196 | T | *Pityogenes chalcographus* | Finland | de Beer | HM031563 | - | HM031504 |
| *O. gilletteae* | CMW30680 |  | *Dendroctonus valens* | USA | S.J. Taerum | KF515870 | - | - |
| *O. gilletteae* | CMW 30681 | T | Beetle in funnel trap | Washington | - | MT637200 | - | MT637227 |
| *O. himal-ulmi* | C1183 |  | Ulmus | India | Heybroek | - | - | AF198233 |
| *O. hongxingense* | CXY1905 |  | *Ips subelongatus* | China | Z. Wang | MN896029 | MN896066 | - |
| *O. hongxingense* | CFCC 52695 | T | *Ips subelongatus* | China | Z. Wang | MN896027 | MN896068 | MK748194 |
| *O. hongxingense* | CFCC 52696 |  | *Ips subelongatus* | China | Z. Wang | MN896030 | MN896067 | - |
| *O. ips* | SYPT1 |  | southern yellow pine | USA | S.H. Kim | AY194954 | - | - |
| *O. ips* | CXY1631 |  | - | China | H. Wang | MH324805 | - | - |
| *O. ips* | DKM 2552 |  | pine wood nematode | South Korea | S.H. Kim | FJ012142 | - | - |
| *O. ips* | CMW7075 | T | *I. integer* | USA | C.T. Rumbold | - | - | AY546704 |
| *O. japonicum* | CMW44469 |  | *P. koraiensis* | China | - | MH124280 | - | MH144084 |
| *O. japonicum* | CMW44468 |  | *P. koraiensis* | China | - | MH124279 | - | MH144083 |
| *O. japonicum* | CMW44592 |  | *P. koraiensis* | China | - | MH124282 | MH124365 | MH144086 |
| *O. japonicum* | YCC099 |  | *Ips subelongatus* invading Japanese larch logs | Japan | T.-C. Lin | - | - | GU134169 |
| *O. jiamusiensis* | CMW40512 | T | *Picea* sp. | Jilin | - | MH124258 | MH124343 | MH144064 |
| *O. karelicum* | 29GRJ |  | *Trypodendron lineatum* beetle | Poland | P. Bilanski | JX444682 | - | - |
| *O. karelicum* | CMW23101 | T | *Scolytus ratzeburgi* infesting birch | Finland | Z.W. de Beer | EU443774 | - | EU443757 |
| *O. karelicum* | CMW23099 | T | *Scolytus ratzeburgi* infesting birch | Russia | R. Linnakoski | EU443773 | - | EU443762 |
| *O. kryptum* | DAOM 229701 | T | *Tetropium* sp. | Austria | T. Kirisits | - | - | AY304436 |
| *O. longiconidiatum* | CMW17574 | T | *Terminalia sericea* | South Africa | G. Kamgan & J. Roux | - | - | EF408558 |
| *O. macroclavatum* | MW23115 | T | *Pityogenes chalcographus* | Linnakoski | R. Linnakoski | HM031557 | - | HM031499 |
| *O. macroclavatum* | MW23115 | T | *Pityogenes chalcographus* | Russia | Z.W. de Beer | KU094723 | KU094765 | HM031499 |
| *O. macroclavatum* | CMW41049 |  | *Ips amitinus* | Estonia | H. Solheim | KU094728 | KU094770 | - |
| *O. macrosporum* | CBS 367.53 |  | - | - | H. Francke-Grosmann | - | - | MH857247 |
| *O. manchongi* | CMW41954 | T | - | China | R. Chang | MH124465 | - | MH121662 |
| *O. manchongi* | CMW41872 |  | - | China | R. Chang | MH124464 | - | - |
| *O. minus* | AU58.4 |  | - | Canada, British Columbia | - | - | - | AF234834 |
| *O. montium* | CBS 151.78 |  | Gallery of *D. ponderosae* in ponderosa pine | USA | R.W. Davidson | AY194963 | - | - |
| *O. montium* | SS519 |  | *Pinus contorta* wood from *Dendroctonus ponderosae* gallery | Canada | A. Rice | - | - | HQ413639 |
| *O. multiannulatum* | MUCL19062 | T | *Pinus* sp. | USA | M. Villarreal | - | - | AY934512 |
| *O. nigricarpum* | CMW650 | T | *Abies* sp. | USA | RW Davidson | AY280479 | - | AY280489 |
| *O. nikkoense* | YCC430 |  | - | Japan | H. Masuya | - | - | AB506674 |
| *O. novo ulmi* | C510 |  | *Ulmus* sp. | USA | - | - | - | AF198236 |
| *O. novo-ulmi* | CMW10573 |  | *Picea abies* | Austria | Neumuller | DQ296095 | - | DQ294375 |
| *O. peniculi* | CFCC52687 | T | - | - | - | MN896035 | MN896063 | MK748198 |
| *O. peniculi* | CFCC52688 | T | - | - | - | MN896034 | MN896061 | - |
| *O. piceae* | C1087 | T | - | Germany | Münch | - | - | AF198226 |
| *O. piceae* | CMW25034 | T | - | Germany | - | KU184312 | KU184398 | KU184441 |
| *O. piceae* | CMW8093 |  | *Tetropium* sp. | Canada | M. YIN | KU184313 | KU184399 | KU184442 |
| *O. piliferum* | CBS129.32 |  | - | Netherlands | S. Schroeder | - | - | AF221070 |
| *O. poligraphi* | CMW38898 |  | *Polygraphus poligraphus* | China | M. YIN | KU184314 | KU184400 | KU184443 |
| *O. poligraphi* | CMW38899 | T | *P. poligraphus* | China | M. YIN | KU184315 | KU184401 | KU184444 |
| *O. pseudobicolor* | CFCC 52684 |  | *Ips subelongatus* | China | Z. Wang | MN896041 | - | MK748190 |
| *O. pseudobicolor* | CFCC 52685 |  | *Ips subelongatus* | China | Z. Wang | MN896039 | - | MK748191 |
| *O. pseudobicolor* | CFCC 52683 | T | *Ips subelongatus* | China | Z. Wang | MN896043 | - | MK748188 |
| *O. pseudocatenulatum* | CBS117571 |  | *I. cembrae* | Atholl, Scotland | T. Kirisits, D. B. Redfern,M.J, Wingfield | KU094734 | KU094771 | HM031500 |
| *O. pseudocatenulatum* | CMW43103 | T | *I. cembrae* | Poland | R. Jankowiak | KU094738 | KU094774 | KU094686 |
| *O. pseudotsugae* | 92-634/302/6 |  | *P. menziesii* infected with *Dendroctonus frontalis* | Canada | C. Goeton | AY548744 | - | AY542502 |
| *O. pulvinisporum* | CMW9022 | T | *Pinus pseudostrobus* | Mexico | - | DQ296100 | - | AY546714 |
| *O. pulvinisporum* | CMW9020 |  | spruce-colonising *Dendroctonus ponderosae* | Canada and Europe | S.M. Alamouti | EU977487 | - | DQ294380 |
| *O. quercus* | CMW19214 |  | - | Norway | - | MH248471 | MH248584 | MH248699 |
| *O. quercus* | CMW2464 |  | - | - | T.A. Duong | - | - | KT779265 |
| *O. quercus* | CMW2467 | T | *Quercus* sp. | France | M. Morelet | - | - | AY466626 |
| *O. rachisporum* | CMW23272 | T | *P. sylvestris* | Finland | de Beer | HM031490 | - | HM031490 |
| *O. sejunctum* | Ophi 1A | T | - | Spain | - | - | - | AY934519 |
| *O. shangrilae* | CMW38900 | T | *Picea purpurea* | China | - | KU184324 | KU184410 | KU184453 |
| *O. shangrilae* | CMW38901 |  | *P. purpurea* | China | - | KU184325 | KU184411 | KU184454 |
| *O. shennongensis* | CFCC53922 |  | *Pinus armandii* | China | H.M. Wang | MW741823 | - | MW459990 |
| *O. shennongensis* | CFCC53921, CXY2501 | T | *Pinus armandii* | China | H.M. Wang | MW741822 | - | MW459989 |
| *O. songshui* | CMW44602 |  | *Ips typographus* on Picea sp. | China | R. Chang, S.F.Chen | MH124260 | MH124345 | MH144066 |
| *O. songshui* | CMW44473 | T | *Ips typographus* on Picea sp. | China | R. Chang, S.F.Chen | MH124259 | MH124344 | MH144065 |
| *O. subalpinum* | MAFF410924 |  | *Polygraphus ssiori* infesting Prunus | Japan | H. Masuya | - | - | AB096211 |
| *O. subelongati* | CFCC52694 |  |  | - | - | MN896054 | MN896065 | - |
| *O. subelongati* | CFCC52693 | T | *Ips subelongatus* infesting *Larix gmelinii* | China | Q. Lu | MN896055 | MN896064 | MH144065 |
| *O. sugadairense* | YCC-588 | T | *Polygraphus kisoensis* in *Larix kaempferi* | Japan | J. Li | AB934353 | LC090747 | LC090226 |
| *O. tapionis* | CMW23266 |  | *Hylastes brunneus* on *Pinus sylvestris* | Finland | R. Linnakoski | HM031544 | KU094778 | - |
| *O. tapionis* | CMW23265 | T | *Pinus sylvestris* | Finland | de Beer | HM031545 | KU094777 | HM031494 |
| *O. tasmaniense* | CMW29088 | T | *Eucalyptus nitens* | - | K. G. Nkuekam | GU797188 | GU797223 | GU797211 |
| *O. tenellum* | CBS189.86 |  | *Pinus pinaster* | USA | M. Villarreal | - | - | AY934523 |
| *O. tetropii* | CBS428.94 |  | *Pinus pinaster* | Australia | M. Villarreal | - | - | AY934524 |
| *O. tingens* | CBS 366.53 | T | - | - | H. Francke-Grosmann | - | - | MH857246 |
| *O. tsotsi* | CMW15239 | T | *Eucalyptus grandis* | Malawi | J.W. Grobbelaar | FJ441280 | FJ441272 | FJ441287 |
| *O. ulmi* | C1182 |  | *Ulmus* | Netherlands | T.C. Harrington | -- | - | AF198232 |
| *O. undulatum* | CMW19396 | T | *Eucalyptus grandis* | South Africa | K.G. Nkuekam | GU797186 | GU797233 | GU797218 |
| *Ophiostoma* sp. 2 | CMW12032 |  | *Pinus* sp. | China | R. Chang | MG205689 | MG205731 | MG205659 |
| *Ophiostoma* sp. B | CMW12032 |  | *Pinus* sp. | China | R. Chang | MG205689 | MG205731 | - |
| *Ophiostoma* sp. 2 | CMW12032 |  | *Pinus* sp. | China | R. Chang | MG205689 | MG205731 | MG205659 |
| *Ophiostoma* sp. B | CMW12032 |  | *Pinus* sp. | China | R. Chang | MG205689 | MG205731 | - |

T = ex-holotype isolate.

ITS: the internal transcribed spacer regions 1 and 2 of the nuclear ribosomal DNA operon, including the 5.8S region;

ITS2-LSU: the internal transcribed spacer 2 and part of the 28S of the rDNA operon;

TUB2: the β-tubulin gene region (TUB2);

TEF1-α: the transcription elongation factor 1-α gene region.

Information missing data are indicated by “-”.
