# Supplementary figures and images for: Identification of ophiostomatalean fungi associated with Tomicus pilifer infesting Pinus koraiensis in Northeastern China
Source: Front Microbiol. 2022 Sep 2;13:919302. doi: 10.3389/fmicb.2022.919302 (PMC9479222; doi:10.3389/fmicb.2022.919302)

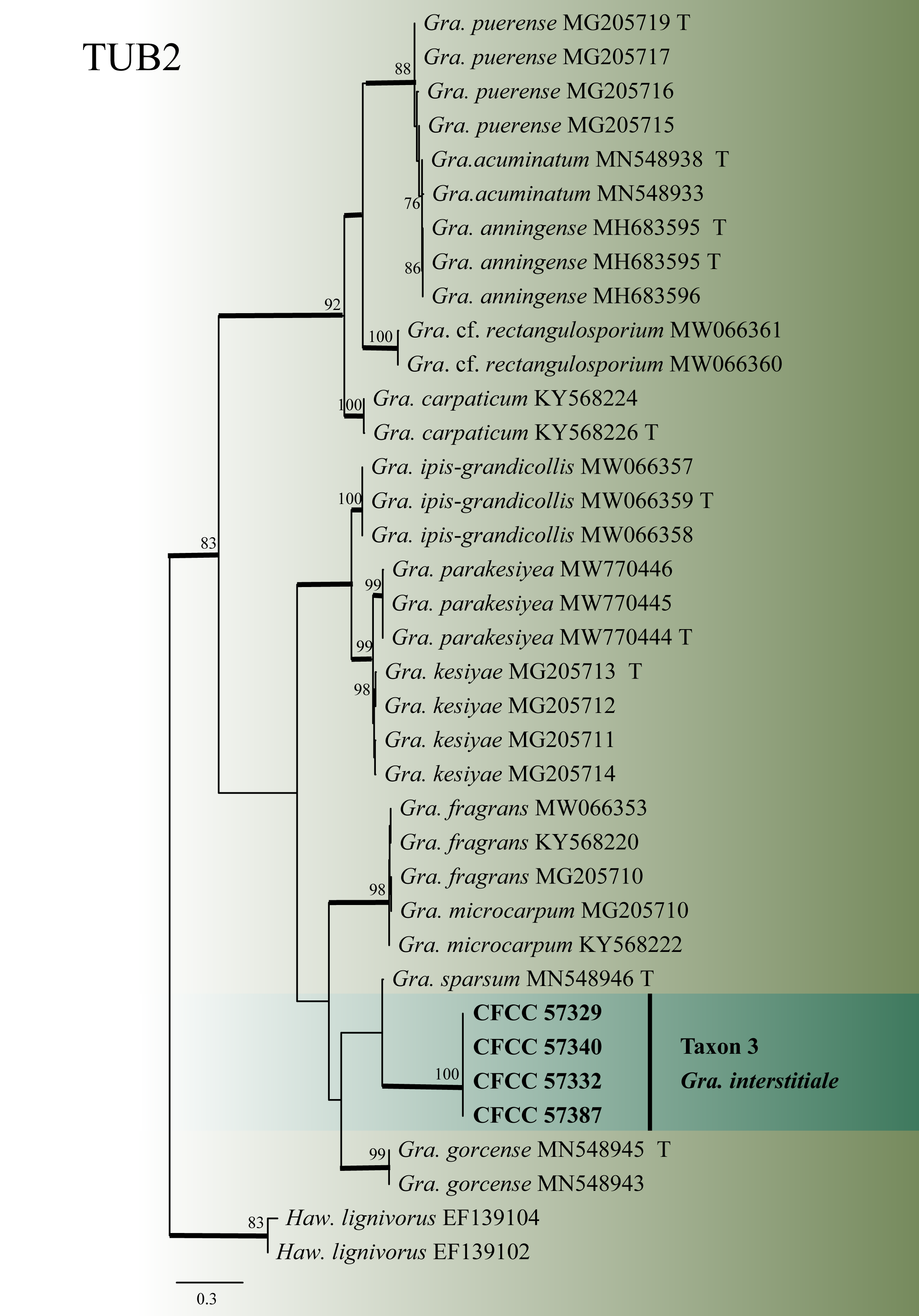

Supplement: Supplementary Figure S1 — ML tree of Graphilbum generated from the TUB2 sequence data. Novel sequences obtained in this study are presented in bold typeface. The bold branches indicate posterior probability values >0.9. Bootstrap values of ML ≥ 70% are recorded at the nodes. T, ex-type strains. The final alignment of 501 positions, including gaps. [file Image_1.TIF]

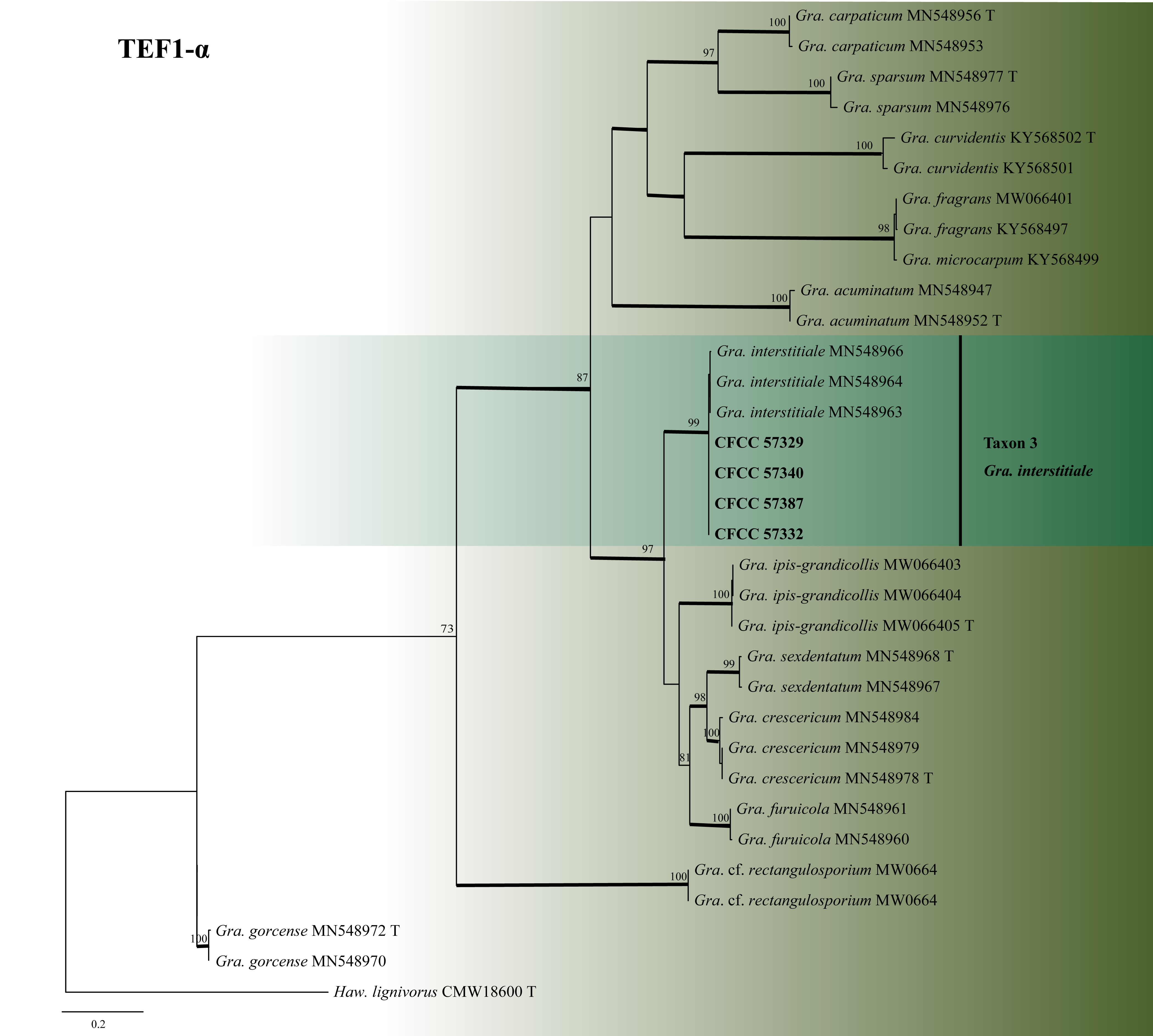

Supplement: Supplementary Figure S2 — ML tree of Graphilbum generated from the EF1-α sequence data. Novel sequences obtained in this study are presented in bold typeface. The bold branches indicate posterior probability values >0.9. Bootstrap values of ML ≥ 70% are recorded at the nodes. T, ex-type strains. The final alignment of 704 positions, including gaps. [file Image_2.TIF]

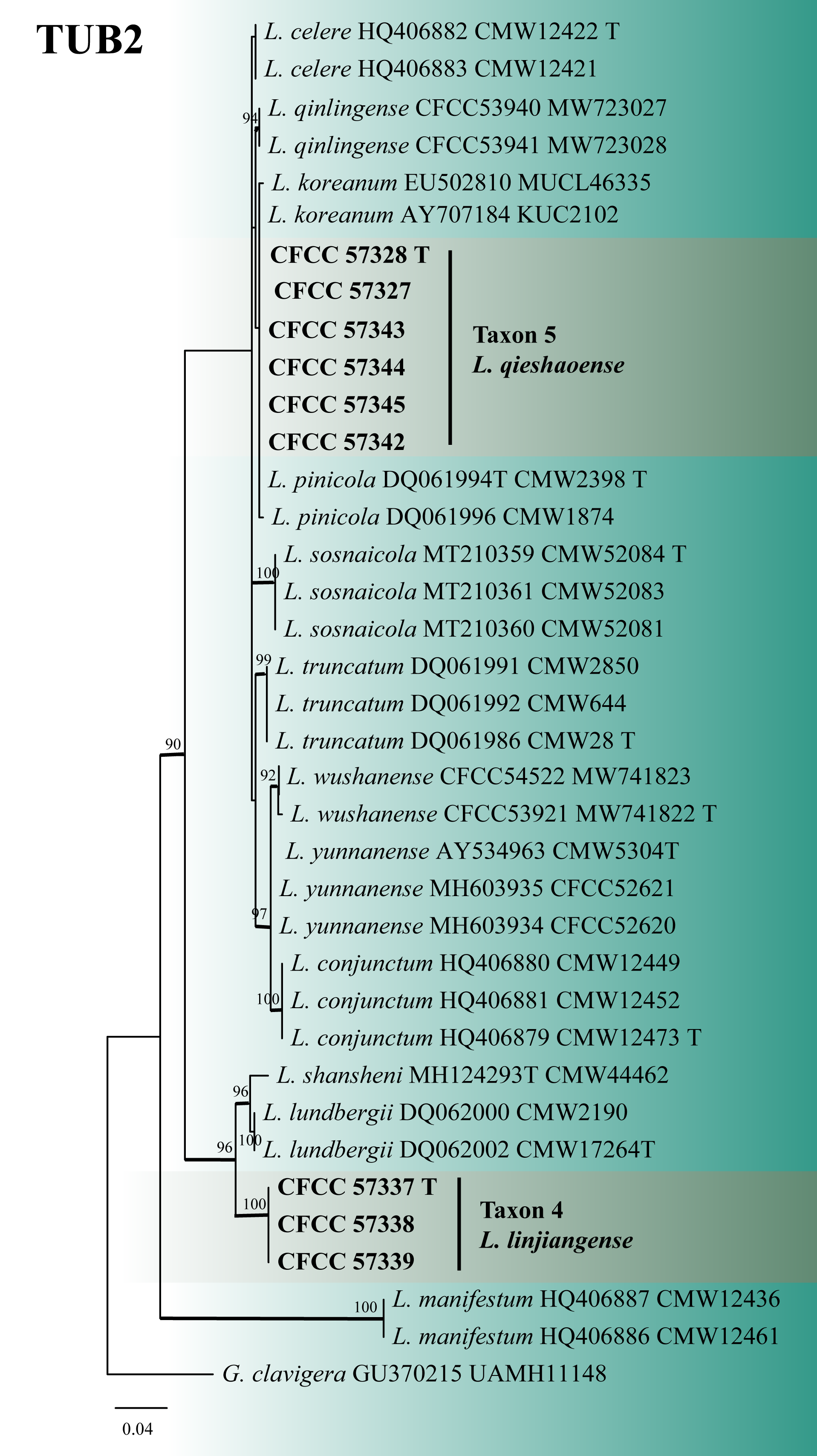

Supplement: Supplementary Figure S3 — ML tree of L. lundbergii complex generated from the TUB2 (Taxon 4, 5) sequence data. Novel sequences obtained in this study are presented in bold typeface. The bold branches indicate posterior probability values >0.9. Bootstrap values of ML ≥ 70% are recorded at the nodes. T, ex-type strains. The final alignment of 369 positions, including gaps. [file Image_3.TIF]

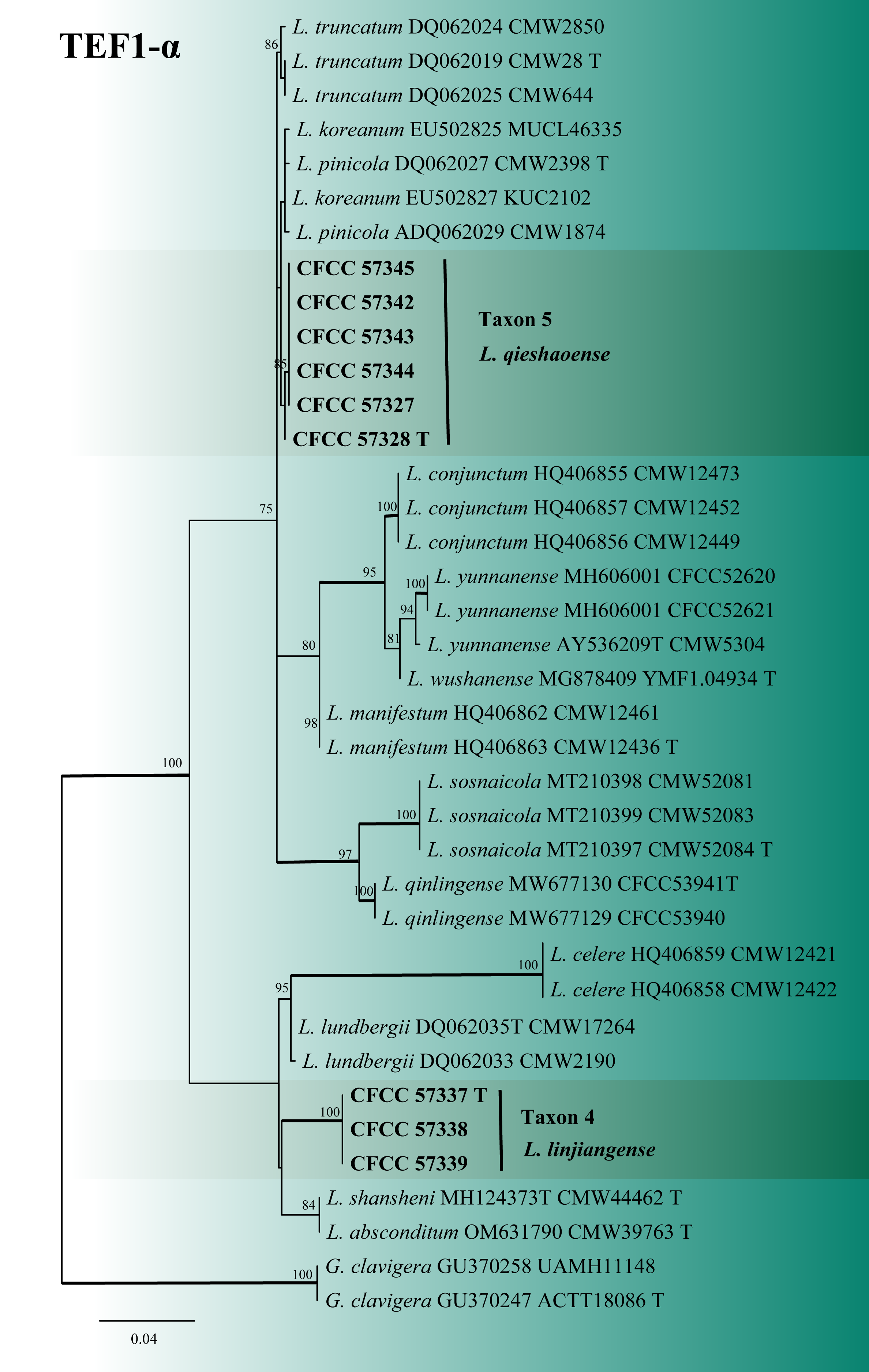

Supplement: Supplementary Figure S4 — ML tree of L. lundbergii complex generated from the EF1-α (Taxon 4, 5) sequence data. Novel sequences obtained in this study are presented in bold typeface. The bold branches indicate posterior probability values >0.9. Bootstrap values of ML ≥ 70% are recorded at the nodes. T, ex-type strains. The final alignment of 635 positions, including gaps. [file Image_4.TIF]

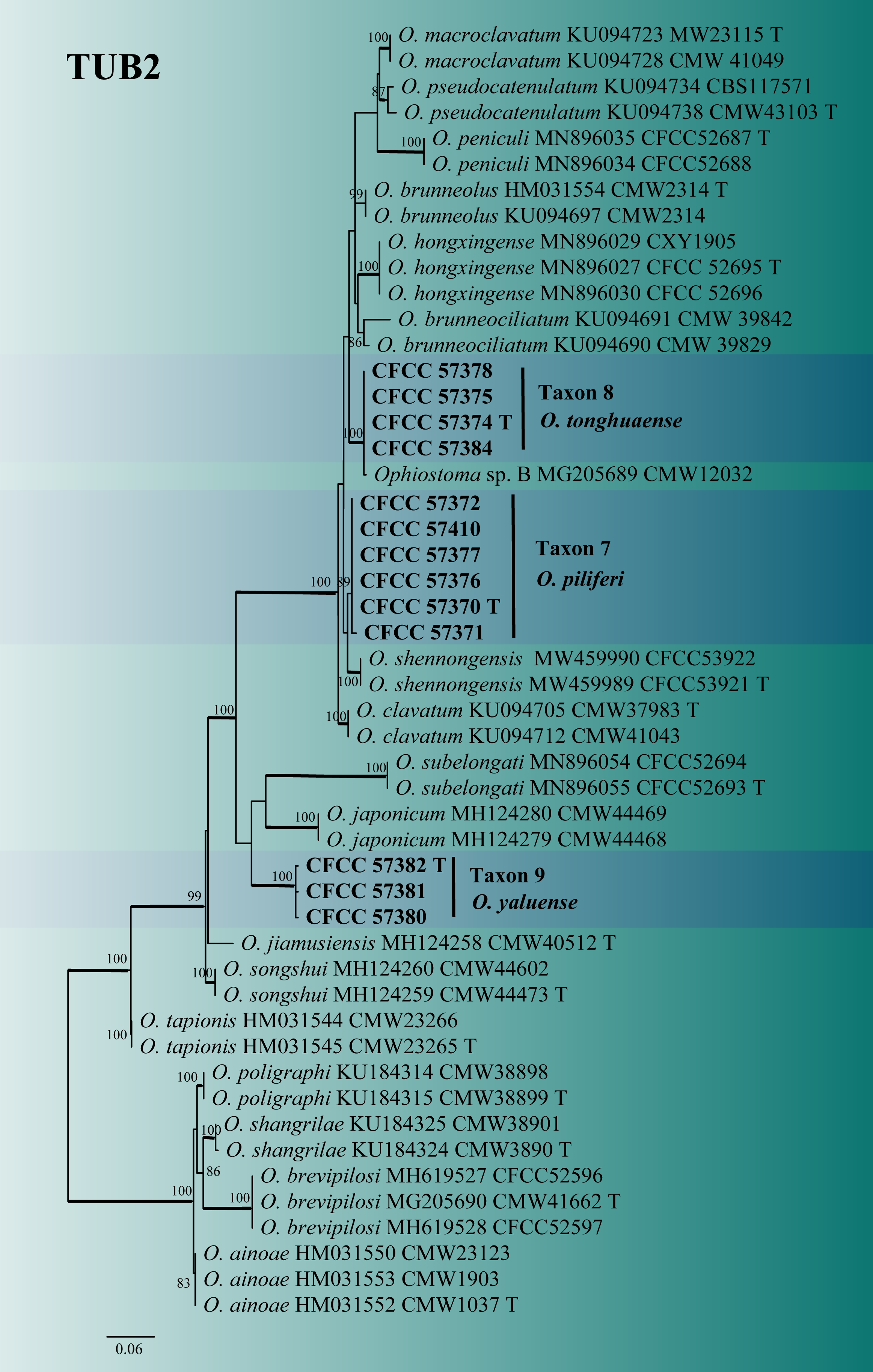

Supplement: Supplementary Figure S5 — ML tree of O. clavatum complex generated from the TUB2 (Taxon 7, 8, 9) sequence data. Novel sequences obtained in this study are presented in bold typeface. The bold branches indicate posterior probability values >0.9. Bootstrap values of ML ≥ 70% are recorded at the nodes. T, ex-type strains. The final alignment of 388 positions, including gaps. [file Image_5.TIF]

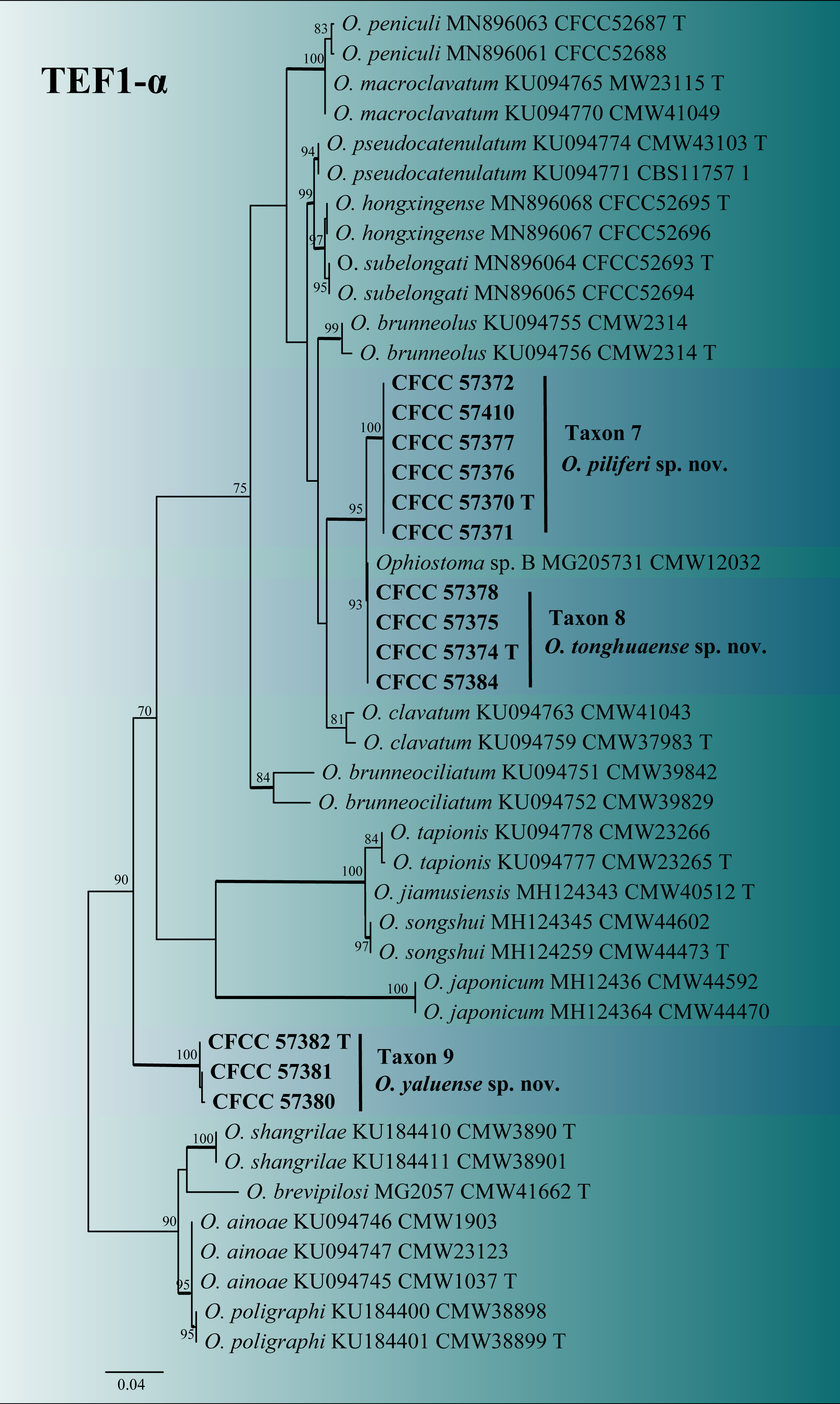

Supplement: Supplementary Figure S6 — ML tree of O. clavatum complex generated from the EF1-α (Taxon 7, 8, 9) sequence data. Novel sequences obtained in this study are presented in bold typeface. The bold branches indicate posterior probability values >0.9. Bootstrap values of ML ≥ 70% are recorded at the nodes. T, ex-type strains. The final alignment of 793 positions, including gaps. [file Image_6.TIF]

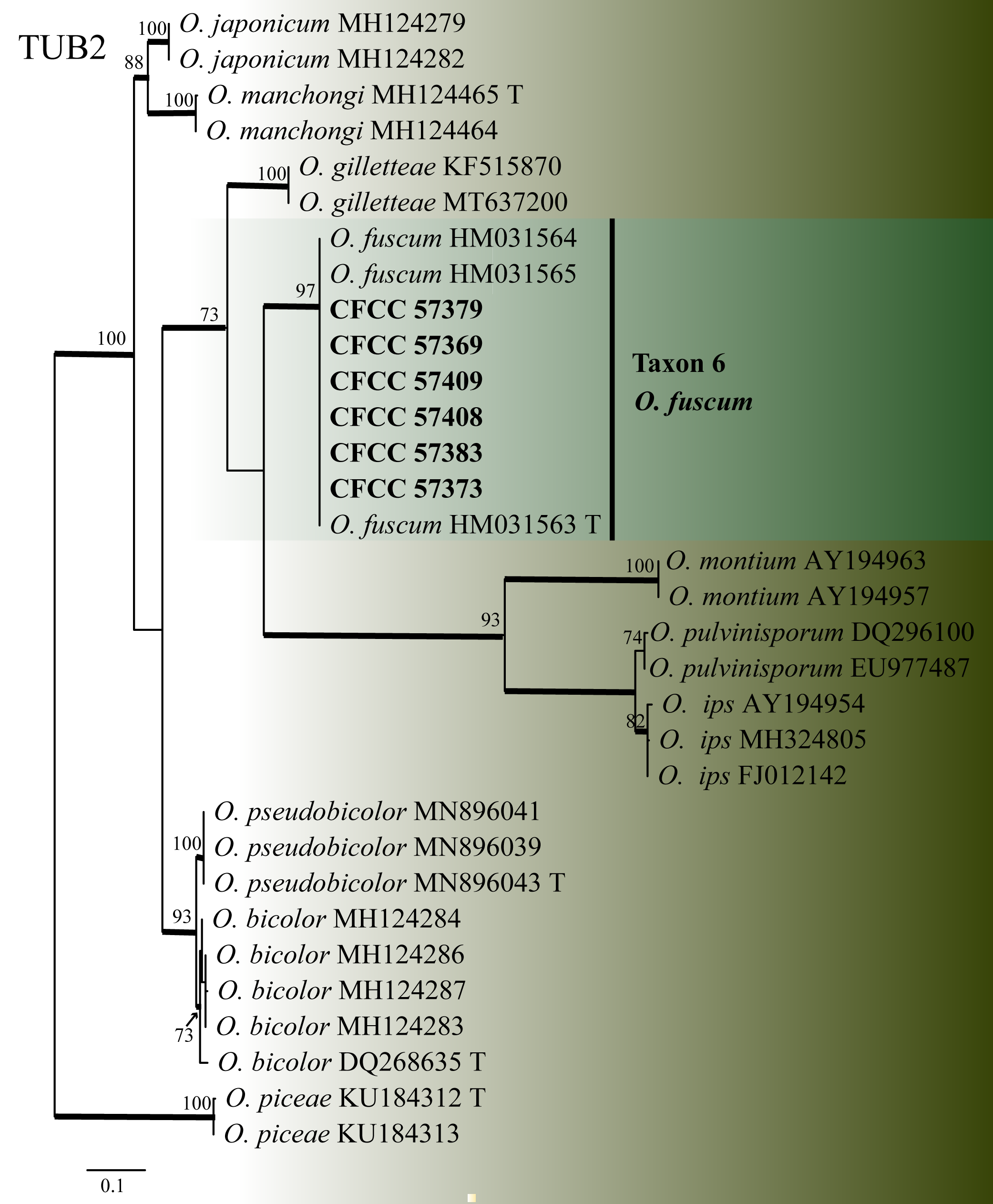

Supplement: Supplementary Figure S7 — ML tree of O. ips complex generated from the TUB2 (Taxon 6) sequence data. Novel sequences obtained in this study are presented in bold typeface. The bold branches indicate posterior probability values >0.9. Bootstrap values of ML ≥ 70% are recorded at the nodes. T, ex-type strains. The final alignment of 414 positions, including gaps. [file Image_7.TIF]
